# A Novel Synthetic Pathway to Lanthanide Triple‐Decker Complexes: Selective Expansion of a Sandwich Complex by an Insertion Reaction

**DOI:** 10.1002/anie.202503942

**Published:** 2025-04-14

**Authors:** Cedric Uhlmann, Luca Münzfeld, Adrian Hauser, Sebastian Gillhuber, Pauline Hädinger, Maxim Leskov, Florian Weigend, Peter W. Roesky

**Affiliations:** ^1^ Institute of Inorganic Chemistry Karlsruhe Institute of Technology (KIT) Kaiserstraße 12 Karlsruhe Germany; ^2^ Institute for Quantum Materials and Technologies Karlsruhe Institute of Technology (KIT) Kaiserstraße 12 Karlsruhe Germany; ^3^ Institute of Nanotechnology (INT) Karlsruhe Institute of Technology (KIT) Kaiserstraße 12 Karlsruhe Germany

**Keywords:** Cyclocene, Insertion reaction, Multi decker, Rare earth, Sandwich complexes

## Abstract

Commonly, multi‐decker sandwich complexes can either be formed in a one‐step reaction or in a stepwise fashion by stacking deck by deck. Herein, we showcase a new reaction pathway, in which for the first time a lanthanide half‐sandwich unit inserts into an existing sandwich complex. As a result of the insertion of a cyclocene half‐sandwich fragment {Ln^II^(*η* ^8^‐Cot^TIPS^)} (Cot^TIPS^ = 1,4‐(*^i^* Pr_3_Si)_2_C_8_H_6_^2−^) into a classical divalent sandwich complex [Ln^II^(*η* ^9^‐Cnt)_2_] (Ln = Sm, Eu, Yb; Cnt = C_9_H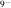), the unprecedented triple‐decker sandwich complexes of the type [(*η* ^9^‐Cnt)Ln^II^(*μ*‐*η* ^8^ *:η* ^8^‐Cot^TIPS^)Ln^II^(*η* ^9^‐Cnt)] were obtained. A plausible reaction pathway was determined by quantum chemical calculations. Additionally, we synthesize the same lanthanide sandwich complexes in a traditional, stepwise fashion. For this, we initially present the novel inverse sandwich compounds [Ln^II^I(thf)_2_(*μ*‐*η* ^8^ *:η* ^8^‐Cot^TIPS^)Ln^II^I(thf)_2_] (Ln = Sm, Eu, thf = tetrahydrofuran) and [Yb^II^(BH_4_)(thf)_2_(*μ*‐*η* ^8^ *:η* ^8^‐Cot^TIPS^)Yb^II^(BH_4_)(thf)_2_] consisting of a cyclooctatetraene middle deck sandwiched between two divalent lanthanides as precursors. Subsequent salt metathesis reactions with [K(Cnt)] (Cnt = C_9_H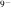) gave rise to the title compounds [(*η* ^9^‐Cnt)Ln^II^(*μ*‐*η* ^8^ *:η* ^8^‐Cot^TIPS^)Ln^II^(*η* ^9^‐Cnt)]. The unique feature of these compounds is the combination of the two largest aromatic all‐carbon rings known in coordination chemistry—the 8‐ and 9‐membered rings—into lanthanide triple‐decker sandwich compounds.

## Graphical abstract

A new reaction pathway is disclosed, in which for the first time a lanthanide half‐sandwich unit inserts into an existing sandwich complex. As a result, a triple‐decker complex is formed, which can also be accessed in a salt metathesis from unprecedented inverse sandwich compounds.

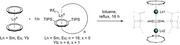

## Introduction

**Figure 1:**
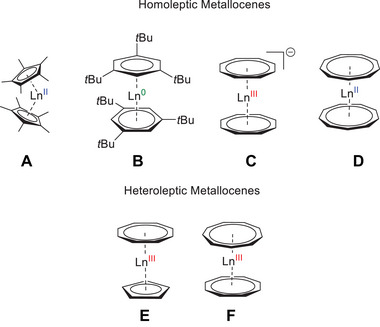
Overview of metallocenes. Selected examples of homo‐ (top) and heteroleptic (bottom) lanthanide sandwich complexes: [Ln^II^(Cp^*^)_2_] (Cp* = C_5_Me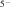, **A**),^[^ ^20^, ^21^, ^22^ ^]^ [Ln^0^(1,3,5‐C_6_H_3_‐*^t^* Bu_3_)_2_] (**B**),^[^ ^5^, ^7^ ^]^ [Ln^III^(Cot)_2_]^−^ (Cot = C_8_H_8_^2−^, **C**),^[^ ^23^, ^24^, ^25^, ^26^, ^27^ ^]^ [Ln^II^(Cnt)_2_] (Cnt = C_9_H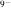, **D**),^[^ ^16^, ^17^ ^]^ [Ln^III^(Cot)(Cp)] (Cp = C_5_H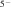, **E**),^[^ ^28^, ^29^ ^]^ [Ln^III^(Cot)(Cnt)] (**F**).^[^ ^18^, ^30^, ^31^ ^]^

Sandwich complexes represent a fundamental class of compounds within organometallic chemistry. Classically, a single metal atom is exclusively coordinated by two aromatic ligands. Homoleptic lanthanide‐based sandwich complexes have successfully been synthesized with a range of ligands, including 5 (**A**),^[^ ^1^, ^2^, ^3^, ^4^ ^]^ 6 (**B**),^[^ ^5^, ^6^, ^7^, ^8^, ^9^, ^10^, ^11^, ^12^ ^]^ 8 (**C**),^[^ ^13^, ^14^, ^15^ ^]^ and most recently, 9‐membered rings (**D**)^[^ ^16^, ^17^, ^18^ ^]^ with respect to purely carbon‐based aromatic monocycles (Figure 1).^[^ ^19^ ^]^ Notably, these ligands, with the exception of the 6‐membered ring, have been employed not only for the construction of homoleptic sandwich complexes but also for the development of heteroleptic congeners (Figure 1, **E–F**).

By formally adding more decks to a sandwich complex, triple‐, quadruple‐, and multi‐decker complexes are obtained.^[^ ^23^ ^]^ In this context, we could recently unveil a new class of sandwich compounds, so‐called “cyclocenes”.^[^ ^32^ ^]^ These species resemble formal 18‐decker [*cyclo*‐M^II^(*μ*‐*η* ^8^ *:η* ^8^‐Cot^TIPS^)]_18_ (M = Sr, Sm, Eu; Cot^TIPS^ = 1,4‐(*^i^* Pr_3_Si)_2_C_8_H_6_^2−^) or quadruple‐decker [*cyclo*‐Yb^II^(*μ*‐*η* ^2^ *:η* ^8^‐Cot^TIPS^)(thf)]_4_ (thf = tetrahydrofuran) compounds forming closed rings in the solid state due to unidirectional bending of each sub‐unit.^[^ ^32^ ^]^

**Figure 2:**
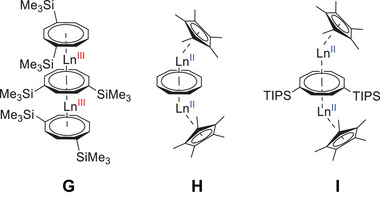
Selected examples of lanthanide triple‐decker complexes: [Ln^III^_2_(Cot″)_3_] (**G**),^[^ ^34^, ^35^, ^36^, ^37^, ^38^ ^]^ [(Cp*)Ln^II^(*μ*‐*η* ^8^ *:η* ^8^‐Cot)Ln^II^(Cp*)] (**H**),^[^ ^39^, ^40^, ^41^ ^]^ and [(Cp*)Ln^II^(*μ*‐*η* ^8^ *:η* ^8^‐Cot^TIPS^)Ln^II^(Cp*)] (**I**)^[^ ^42^ ^]^ (TIPS = triisopropylsilyl).

The most intensely studied lanthanide multi‐deckers are triple‐decker complexes, featuring both trivalent and divalent lanthanides. Notable examples include the bent and asymmetric [(*η* ^8^‐Cot)Ln^III^(*μ*‐*η* ^8^:*η* ^2^‐Cot)Ln^III^(thf)_2_(*η* ^8^‐Cot)] (Ln = La, Ce, Nd, Er)^[^ ^33^ ^]^ and linear complexes incorporating substituted Cot ligands of the type [Ln^III^_2_(*η* ^8^‐Cot´´)_3_] (Cot´´ = 1,4‐(Me_3_Si)_2_C_8_H_6_^2−^; Ln = La, Ce, Nd, Sm, Tb, Ho, Er, Tm, Lu) (Figure 2, **G**).^[^ ^34^, ^35^, ^36^, ^37^, ^38^ ^]^ For divalent lanthanides, the charge of two Ln^II^ ions can be compensated by one Cot and two cyclopentadienyl ligands, leading to the formation of heteroleptic species [(*η* ^5^‐Cp*)Ln^II^(*μ*‐*η* ^8^ *:η* ^8^‐Cot)Ln^II^(*η* ^5^‐Cp*)] (Cp* = C_5_Me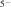; Ln = Sm, Eu, Yb) (Figure 2, **H**)^[^ ^39^, ^40^, ^41^ ^]^ and [(*η* ^5^‐Cp*)Ln^II^(*μ*‐*η* ^8^ *:η* ^8^‐Cot^TIPS^)Ln^II^(*η* ^5^‐Cp*)] (Figure 2, **I**).^[^ ^42^ ^]^ The synthesis of these compounds is traditionally achieved via two different pathways: (1) salt metathesis involving lanthanide halides and alkali metal Cot derivatives^[^ ^15^, ^43^ ^]^ or (2) reductive elimination of a Cot ligand from sandwich compounds, e.g., reaction of [Ln(*η* ^8^‐Cot)_2_]^−^ with CoCl_2_.^[^ ^35^ ^]^ Herein, we report two unprecedented synthetic approaches leading to the first triple‐decker structures incorporating the cyclononatetraenyl ligand, [(*η* ^9^‐Cnt)Ln^II^(*μ*‐*η* ^8^ *:η* ^8^‐Cot^TIPS^)Ln^II^(*η* ^9^‐Cnt)], following two distinct pathways:

1) A fundamental new reaction pathway towards multi‐decker sandwich compounds, specifically the insertion of a cyclocene half‐sandwich fragment {Ln^II^(*η* ^8^‐Cot^TIPS^)} into a classical divalent sandwich complex [Ln^II^(*η* ^9^‐Cnt)_2_].^[^ ^43^, ^44^ ^]^

2) Syntheses of the same triple‐decker compounds by salt metathesis from the first inverse lanthanide sandwich Cot complexes bearing apical (pseudo‐)halide ligands [Ln^II^X(*μ*‐*η* ^8^ *:η* ^8^‐Cot^TIPS^)Ln^II^X] (X = I, BH_4_).^[^ ^43^ ^]^

## Results and Discussion

### Synthesis and Structural Characterization

**Scheme 1:**
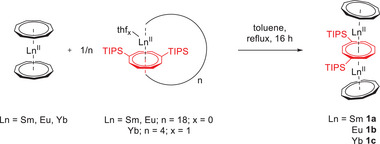
Formation of lanthanide triple‐decker compounds by insertion. Synthesis of **1a–c** by insertion of [Ln^II^(*η* ^8^‐Cot^TIPS^)(thf)_x_]_n_ (for Ln = Sm, Eu; *n* = 18; *x* = 0 and for Ln = Yb; *n* = 4; *x* = 1) into [Ln^II^(*η* ^9^‐Cnt)_2_] (TIPS = triisopropylsilyl).

Upon synthesis of the cyclocenes, we realized that they can be reversibly transformed from their mononuclear half‐sandwich form [M^II^(thf)_3_(*η* ^8^‐Cot^TIPS^)] to the corresponding cyclocenes and back, depending on the solvent and the reaction conditions.^[^ ^32^, ^43^ ^]^ For compound [*cyclo*‐Yb^II^(*μ*‐*η* ^2^ *:η* ^8^‐Cot^TIPS^)(thf)]_4_ in the weakly‐coordinating solvent toluene, the hydrodynamic radii determined by DOSY NMR indicated the formation of a monomeric species in solution.^[^ ^32^ ^]^ Thus, in the absence of any donor solvent, a highly reactive half‐sandwich species {Ln^II^(*η* ^8^‐Cot^TIPS^)} is expected to be present in solution. Aiming at investigating the reactivity of this species, 1/*n* [*cyclo*‐Ln^II^(*η* ^8^‐Cot^TIPS^)]_n_ (*n* = 18 for Ln = Sm, Eu, and *n* = 4 for Ln = Yb) was treated with the classical sandwich compounds [Ln^II^(*η* ^9^‐Cnt)_2_].^[^ ^16^, ^17^, ^43^ ^]^ After 16 h of stirring in refluxing toluene, workup, and recrystallization from hot toluene, single crystals suitable for X‐ray structural analysis of the heteroleptic triple‐decker sandwich complexes [(*η* ^9^‐Cnt)Ln^II^(*μ*‐*η* ^8^ *:η* ^8^‐Cot^TIPS^)Ln^II^(*η* ^9^‐Cnt)] (Cnt = C_9_H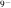) (**1a** = Sm, **1b **= Eu, **1c** = Yb) were isolated (Scheme 1). This confirms that a formal half‐sandwich fragment of the type {Ln^II^(*η* ^8^‐Cot^TIPS^)} can indeed be employed for insertion into an existing sandwich system. This up‐to‐date unknown reactivity within the field of lanthanide chemistry expands the synthetic possibilities for constructing lanthanide multi‐decker compounds. Compounds **1a–c** are the first lanthanide triple‐decker species with purely carbon‐based ring systems featuring apical ligand systems other than Cp or Cot derivatives.^[^ ^45^ ^]^

**Figure 3:**
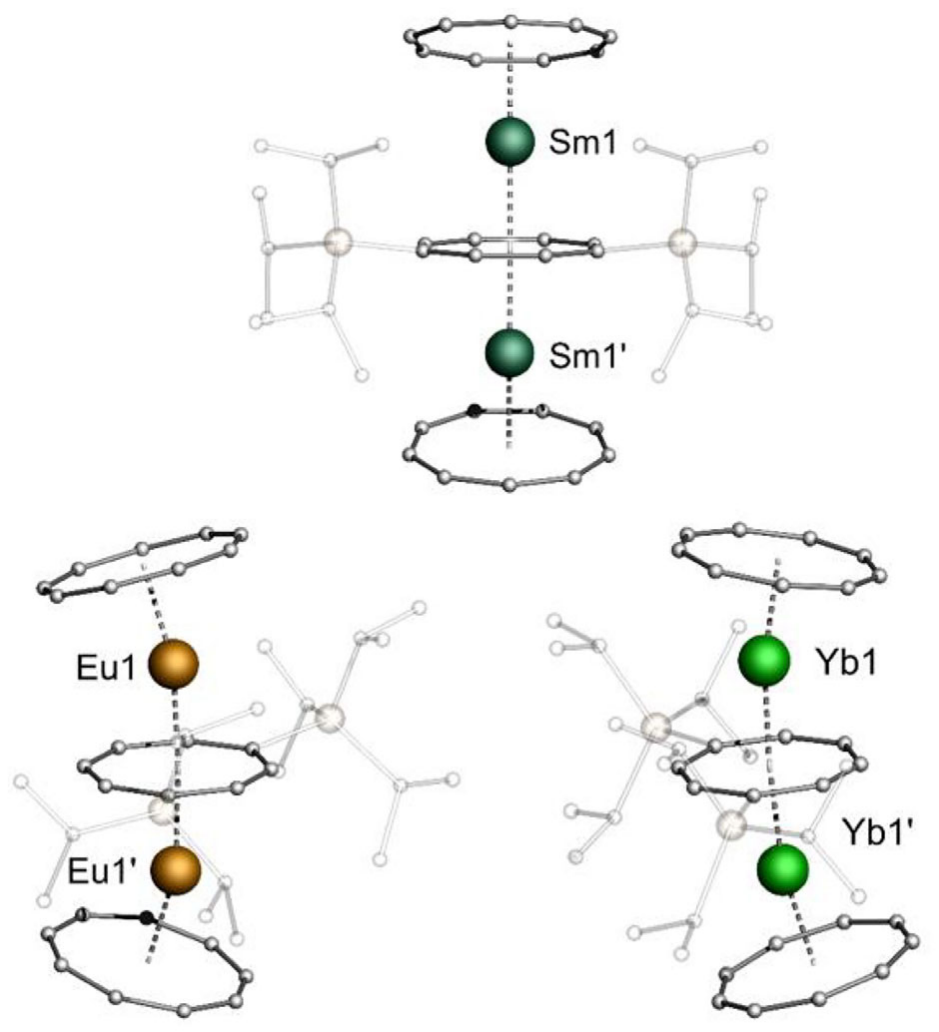
[(*η* ^9^‐Cnt)Ln^II^(*μ*‐*η* ^8^:*η* ^8^‐Cot^TIPS^)Ln^II^(*η* ^9^‐Cnt)]. Molecular structures of **1a** (top), **1b** (bottom left), and **1c** (bottom right) in the solid state. Only one part of the disordered Cnt ligands and TIPS groups of **1a** and **1b** is shown. Hydrogen atoms are not shown, and TIPS groups are depicted transparently for clarity. Color code: C: light gray. For detailed bond lengths and angles, refer to the Supporting Information (Tables S3–S5).

In the solid state, **1a–c** form the anticipated triple‐decker structural motif with *η* ^9^‐coordinated Cnt outer decks and a *μ*‐*η* ^8^ *:η* ^8^‐bridging Cot^TIPS^ middle deck (Figure 3). All compounds are structurally isomorphic. Notably, **1a** and **1b** show some disorder of the TIPS groups and the Cnt rings, which is not observed for **1c**. Consistent with lanthanide contraction, the Ln1─Ct_Cot_ (Ct = centroid of the corresponding ring) distance is 2.1551(4) Å for **1a**, 2.1505(4) Å for **1b**, and 2.1009(6) Å for **1c**. Compared to the Cp* capped analogues [(*η* ^5^‐Cp*)Ln^II^(*μ*‐*η* ^8^ *:η* ^8^‐Cot^TIPS^)Ln^II^(*η* ^5^‐Cp*)], these values are slightly elongated, probably due to the higher steric demand of the Cnt ligand.^[^ ^42^ ^]^ For the Ln1─Ct_Cnt_ distances, a similar trend is observed along the series with 2.0949(4) Å (**1a**), 2.1004(4) Å (**1b**), and 2.0333(6) Å (**1c**). Notably, the molecules are strongly bent with Ct_Cnt_─Ct_Cot_─Ct_Cnt_ angles of 159.935(2)° (**1a**), 160.324(2)° (**1b**), and 165.157(2)° (**1c**), respectively. While the inner [Ln_2_^II^(*μ*‐*η* ^8^ *:η* ^8^‐Cot^TIPS^)]^2+^ fragments with Ln1─Ct_Cot_─Ln1' angles of about 176° are almost linear; the outer [(*η* ^8^‐Cot^TIPS^)Ln^II^(*η* ^9^‐Cnt)]^−^ fragments with Ct_Cot_─Ln1─Ct_Cnt_ angles of 163.86(2)° (**1a**), 164.24(2)° (**1b**), and 169.50(3)° (**1c**) show significant deviations from linearity. The bending occurs away from the TIPS substituents and is presumably induced by their high steric demand. The convergence to a linear orientation along the row can be explained by increasing steric pressure in the systems with decreasing ionic radii.

In addition to the structural characterization of the compounds in the solid state by single crystal X‐ray diffraction analysis, NMR spectroscopic investigations were carried out in THF‐*d*_8_. For **1a** and **1c**, all expected resonances were detected. For **1b**, the strongly paramagnetic character of the compound prevented the collection of meaningful NMR data. Worth noting is the resonance of the 18 magnetically equivalent Cnt protons of the paramagnetic compound **1a**. This is seen at a chemical shift of *δ* = 18.48 ppm—a value close to the chemical shift of the Cnt ligand in [Sm^II^(*η* ^9^‐Cnt)_2_] (*δ* = 20.67 ppm in C_6_D_6_).^[^ ^17^ ^]^ Interestingly, compound **1b** was not found to exhibit any photoluminescence (PL). This is in sharp contrast to [Eu^II^(Cnt)_2_], where the Cnt ligands induce interesting PL excitation (PLE) properties.^[^ ^16^, ^43^ ^]^

In a second approach, we aimed at synthesizing **1a–c** in a classical approach by piling up the decks, leading to the triple‐decker complexes **1a–c** in a stepwise fashion. However, no suitable precursor to accomplish this has previously been reported in the literature. Therefore, we initially aimed at synthesizing inverse sandwich complexes of the general formula [Ln^II^X(*μ*‐*η* ^8^ *:η* ^8^‐Cot^TIPS^)Ln^II^X] (X = halide or pseudohalide), which have not been reported in the literature so far. This is followed by the addition of Cnt ligands to the apical coordination sites in a second step.

**Scheme 2:**
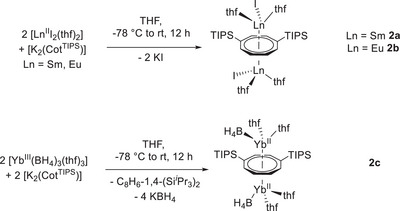
Formation of lanthanide inverse sandwiches by salt elimination. Synthesis of [Ln^II^I(thf)_2_(*μ*‐*η* ^8^ *:η* ^8^‐Cot^TIPS^)Ln^II^I(thf)_2_] (**2a** = Sm, **2b** = Eu) and [Yb^II^(BH_4_)(thf)_2_(*μ*‐*η* ^8^ *:η* ^8^‐Cot^TIPS^)Yb^II^(BH_4_)(thf)_2_] (**2c**) (TIPS = triisopropylsilyl).

For X = I, the target compounds [Ln^II^I(thf)_2_(*μ*‐*η* ^8^ *:η* ^8^‐Cot^TIPS^)Ln^II^I(thf)_2_] (**2a** = Sm, **2b** = Eu) were synthesized via salt elimination of the divalent lanthanide diiodides in THF with the potassium salt [K_2_Cot^TIPS^] (Scheme 2).^[^ ^46^ ^]^ The corresponding Yb compound could not be obtained by this method. However, the analog borohydride complex [Yb^II^(BH_4_)(thf)_2_(*μ*‐*η* ^8^ *:η* ^8^‐Cot^TIPS^)Yb^II^(BH_4_)(thf)_2_] (**2c**) was isolated by reaction of the trivalent precursor [Yb^III^(BH_4_)_3_(thf)_3_] and [K_2_(Cot^TIPS^)] in a 1:1 ratio (Scheme 2). The formation of **2c** seems unexpected at first sight but can be rationalized by in situ reduction of the trivalent Yb precursor by half an equivalent of the present Cot^TIPS^ ligand. The reduction potential of the unsubstituted Cot dianion is *E*_1/2_ = −1.82 to −1.86 V versus SCE (saturated calomel electrode), depending on the source.^[^ ^47^, ^48^ ^]^ Considering the half‐cell potential of the Yb^III^/Yb^II^ redox pair of *E*_1/2_ = −1.15 V, Cot derivatives should in principle be capable of reducing trivalent Yb;^[^ ^49^ ^]^ a phenomenon already reported elsewhere by Edelmann et al.^[^ ^50^ ^]^ Surprisingly, **2c** is not accessible starting from [Yb^II^(BH_4_)_2_(thf)_4_].^[^ ^51^ ^]^

The clean isolation of the reduced material was confirmed by ^171^Yb{^1^H} and ^1^H NMR spectroscopic studies (see Supporting Information Chapter 2). No signs of paramagnetically shifted resonances, which are expected for trivalent Yb species, were observed during the NMR spectroscopic investigations. Moreover, the two singlets found at *δ* = 338.7 ppm and *δ* = 310.5 ppm in the ^171^Yb{^1^H} NMR spectrum are in the range of reported divalent Yb‐BH_4_ species.^[^ ^51^ ^]^ Since the ^1^H, ^11^B, and ^13^C NMR spectra do not indicate the presence of a second Yb species, we suggest some dynamic processes, which could not be resolved by VT NMR studies, may be the reason.

**Figure 4:**
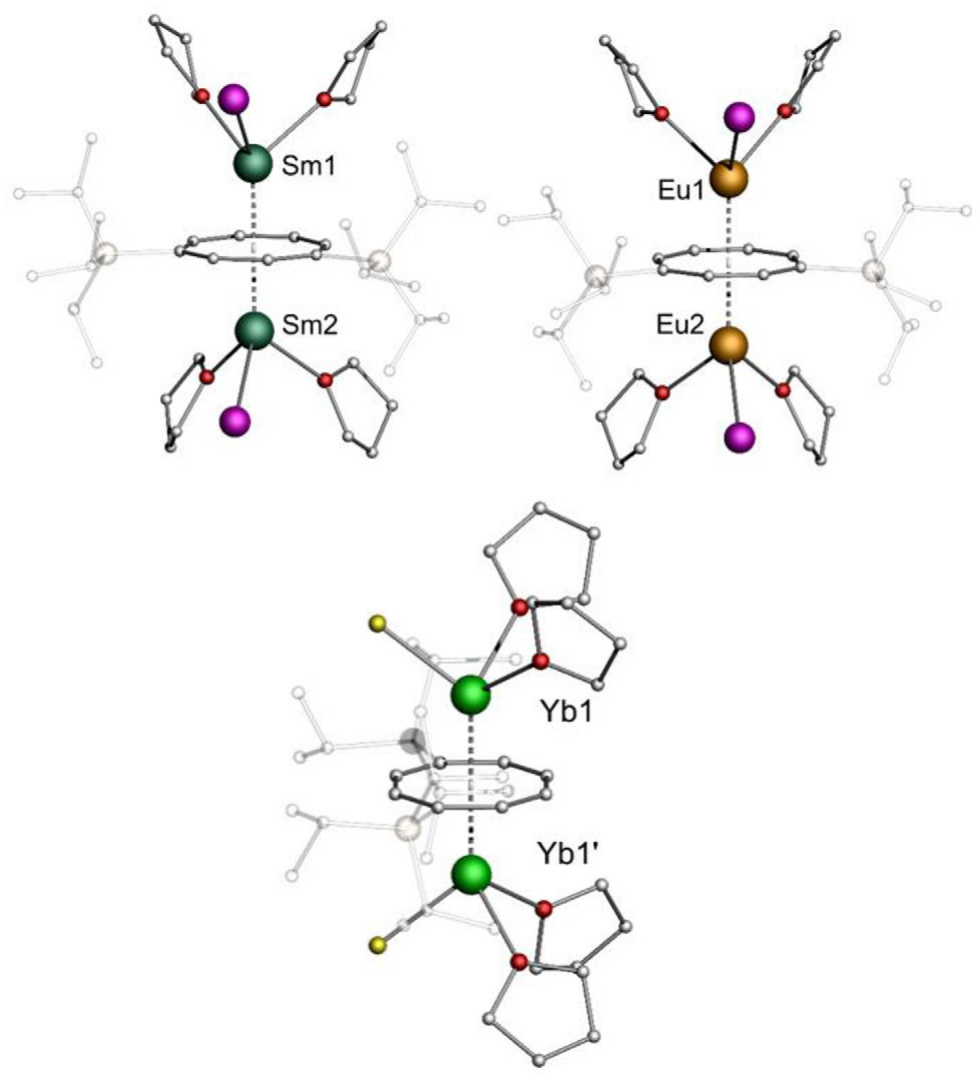
Lanthanide inverse sandwich complexes. Molecular structures of **2a** (top left), **2b** (top right), and **2c** (bottom) in the solid state. Only one part of the statistically disordered THF ligands and TIPS substituents of the compounds is shown. Hydrogen atoms are not shown and TIPS groups are displayed transparently for clarity. Color code: C: light gray, O: red, I: purple, B: yellow. For detailed bond lengths and angles refer to the Supporting Information (Tables S6–S8).

Single crystals of **2a–c** were obtained by recrystallization of the complexes from a hot mixture of toluene and THF (volume ratio 5:1). The coordination sphere of the lanthanide cations of complexes **2a–c** exhibits a piano–stool structural motif with one halide or pseudohalide ion and two neutral THF ligands bound to the central metal ions (Figure 4). The overall molecular structure resembles that of an inverse sandwich (see Supporting Information Chapter 3 for structural details).

Compound **2b** shows, similar to the reported triple‐decker compound [(Cp*)Eu^II^(*μ*‐*η* ^8^ *:η* ^8^‐Cot^TIPS^)Eu^II^(Cp*)],^[^ ^42^, ^52^ ^]^ intense blue‐green luminescence upon UV light (365 nm) excitation at room temperature (Figure S7). The PLE spectrum is characterized by a broad, unstructured absorption band, which can be attributed to the 4f^7^→4f^6^5d^1^ transitions. Similarly, the PL spectrum is characterized by a single, broad band at 501 nm, likely resulting from the corresponding 4f^6^5d^1^→4f^7^ relaxation process.^[^ ^53^, ^54^ ^]^ The lifetime of the process was determined to be 1.13 µs, which is within the expected range for f–d transitions in molecular compounds of divalent Eu.^[^ ^55^, ^56^, ^57^ ^]^

**Scheme 3:**
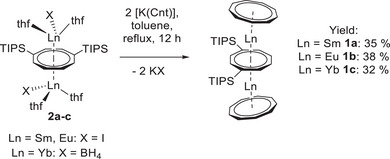
Formation of lanthanide triple‐decker compounds by salt elimination. Synthesis of the Cnt triple‐decker complexes **1a–c** from the inverse sandwich complexes **2a–c** by salt elimination reactions with [K(Cnt)] in toluene (TIPS = triisopropylsilyl).

Having the inverse sandwich complexes **2a–c** at hand, we aimed at replacing the (pseudo‐)halide ligands by the Cnt ligand to generate **1a–c**. While treatment of **2a–c** with [K(Cnt)]^[^ ^58^ ^]^ in THF did not yield the desired products, the reaction proceeds in refluxing toluene (Scheme 3). Compounds **1a–c** were isolated after workup and crystallization in moderate yields and as single crystals suitable for X‐ray structural analysis. The yield of the products of this classical route is slightly lower compared to the insertion route.^[^ ^43^, ^44^ ^]^

### Quantum Chemical Calculations

For a more detailed understanding of the formation of this triple‐decker complex motif via insertion of a half‐sandwich unit into an existing sandwich complex, quantum chemical calculations at the density functional theory (DFT) level were performed with TURBOMOLE.^[^ ^59^, ^60^ ^]^ Calculations were performed with the PBE0 hybrid functional^[^ ^61^, ^62^ ^]^ and def2‐TZVP basis sets,^[^ ^63^ ^]^ unless explicitly noted otherwise. Computational details are given in the Supporting Information (Chapter 8).

Based on the literature,^[^ ^32^ ^]^ it is evident that the cyclocenes, employed for the synthesis of compounds **1a–c** via the pathway depicted in Scheme 1, do not exist as large cyclic structures in toluene solution. Instead, they are expected to dissolve as monomeric half‐sandwich complexes of the type {Ln^II^(*η* ^8^‐Cot^TIPS^)}. In principle, reaction of the latter with [Ln^II^(*η* ^9^‐Cnt)_2_] could result in two conceivable structural motifs. Either the experimentally observed [(*η* ^9^‐Cnt)Ln^II^(*μ*‐*η* ^8^ *:η* ^8^‐Cot^TIPS^)Ln^II^(*η* ^9^‐Cnt)], featuring a bridging Cot^TIPS^ ligand sandwiched between two [Ln^II^(*η* ^9^‐Cnt)]^+^ fragments, or [(*η* ^9^‐Cnt)Ln^II^(*μ*‐*η* ^9^:*η* ^9^‐Cnt)Ln^II^(*η* ^8^‐Cot^TIPS^)], featuring a bridging Cnt ligand sandwiched by one [Ln^II^(*η* ^9^‐Cnt)]^+^ and one {Ln^II^(*η* ^8^‐Cot^TIPS^)} fragment.

Comparison of the formation energies of [(*η* ^9^‐Cnt)Ln^II^(*μ*‐*η* ^8^ *:η* ^8^‐Cot^TIPS^)Ln^II^(*η* ^9^‐Cnt)] and [(*η* ^9^‐Cnt)Ln^II^(*μ*‐*η* ^9^:*η* ^9^‐Cnt)Ln^II^(*η* ^8^‐Cot^TIPS^)] from the starting materials [Ln^II^(*η* ^9^‐Cnt)_2_] and [Ln^II^(*η* ^8^‐Cot^TIPS^)] reveals that the experimentally observed structures are energetically preferred by 117 kJ mol^−1^ (Sm), 116 kJ mol^−1^ (Eu), and 112 kJ mol^−1^ (Yb), respectively, over the conceivable alternatives (refer to Supporting Information Figure S28).

Indeed, this difference in formation energy can be rationalized by a simplified reasoning solely based on electrostatic arguments (refer to Supporting Information Tables S9 and S10 for details). Considering the natural population analysis (NPA)^[^ ^64^ ^]^ charges of the Ln ions and the individual ligands as point charges located at the positions of the lanthanide ions and in the ring centroid of each cyclic ligand gives a difference in electrostatic interaction energy of 254 kJ mol^−1^ (Sm), 252 kJ mol^−1^ (Eu), and 150 kJ mol^−1^ (Yb), respectively, in favor of the experimentally observed [(*η* ^9^‐Cnt)Ln^II^(*μ*‐*η* ^8^ *:η* ^8^‐Cot^TIPS^)Ln^II^(*η* ^9^‐Cnt)] over the unfavorable alternative [(*η* ^9^‐Cnt)Ln^II^(*μ*‐*η* ^9^:*η* ^9^‐Cnt) Ln^II^(*η* ^8^‐Cot^TIPS^)].

Apart from electrostatic interactions, additional contributions to the difference in formation energies arise from dispersion interactions, which also differ slightly for the constitutional isomers under consideration. Dispersion interactions are higher by 32 kJ mol^−1^ (Sm), 33 kJ mol^−1^ (Eu), and 34 kJ mol^−1^ (Yb), respectively, for structures featuring a bridging Cot^TIPS^ ligand than for structures with a bridging Cnt moiety.

Regarding the formation of the experimentally observed structural motif, several fundamentally distinct pathways seem conceivable (refer to Supporting Information Figure S30): (1) One Cnt ligand could dissociate from the starting material, yielding a solution of [Ln^II^(*η* ^9^‐Cnt)]^+^, Cnt^−^, and [Ln^II^(*η*‐Cot^TIPS^)], subsequently forming the thermodynamically preferred arrangement, [(*η* ^9^‐Cnt)Ln^II^(*μ*‐*η* ^8^ *:η* ^8^‐Cot^TIPS^)Ln^II^(*η* ^9^‐Cnt)]. (2) [Ln^II^(*η* ^9^‐Cnt)_2_] and [Ln^II^(*η* ^8^‐Cot^TIPS^)] could initially form the thermodynamically less preferred [(*η* ^9^‐Cnt)Ln^II^(*μ*‐*η* ^9^:*η* ^9^‐Cnt)Ln^II^(*η* ^8^‐Cot^TIPS^)], which subsequently splits into [Ln^II^(*η* ^8^‐Cot^TIPS^)(*η* ^9^‐Cnt)]^−^ and [Ln^II^(*η* ^9^‐Cnt)]^+^. The resulting fragments could then recombine to the thermodynamically preferred product [(*η* ^9^‐Cnt)Ln^II^(*μ*‐*η* ^8^ *:η* ^8^‐Cot^TIPS^)Ln^II^(*η* ^9^‐Cnt)]. (3) The [Ln^II^(*η* ^8^‐Cot^TIPS^)] fragment could approach the Ln ion in [Ln^II^(*η* ^9^‐Cnt)_2_] with the negatively polarized Cot^TIPS^ site, followed by a rearrangement to the observed product in a concerted fashion.

For option (1), calculations of the dissociation energy of [Ln^II^(*η* ^9^‐Cnt)_2_] to [Ln^II^(*η* ^9^‐Cnt)]^+^ and Cnt^−^ using the dielectric constant of toluene (*ε* = 2.4) yield 381 kJ mol^−1^ (Sm), 376 kJ mol^−1^ (Eu), and 389 kJ mol^−1^ (Yb), respectively, rendering this pathway highly unlikely.

Similarly, for option (2), calculations of the dissociation energy of [(*η* ^9^‐Cnt)Ln^II^(*μ*‐*η* ^9^:*η* ^9^‐Cnt)Ln^II^(*η* ^8^‐Cot^TIPS^)] to [Ln^II^(*η* ^9^‐Cnt)]^+^ and [Ln^II^(*η* ^8^‐Cot^TIPS^)(*η* ^9^‐Cnt)]^‐^ using the dielectric constant of toluene (*ε* = 2.4) yield 277 kJ mol^−1^ (Sm), 273 kJ mol^−1^ (Eu), and 298 kJ mol^−1^ (Yb), respectively, rendering this pathway also unfeasible under the employed reaction conditions.

**Figure 5:**
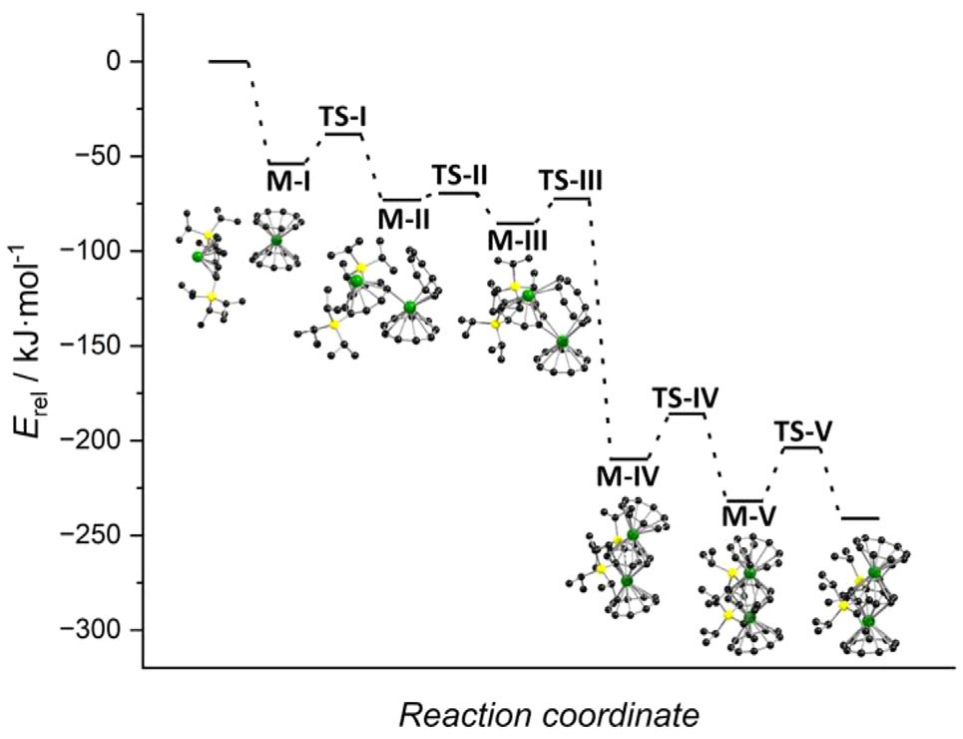
Calculated insertion pathway. Energies of the minima and transition states (PBE0/def2‐SV(P)/D4) along the reaction path of the insertion of {Yb^II^(*η* ^8^‐Cot^TIPS^)} into [Yb^II^(*η* ^9^‐Cnt)_2_] to form [(*η* ^9^‐Cnt)Yb^II^(*μ*‐*η* ^8^ *:η* ^8^‐Cot^TIPS^)Yb^II^(*η* ^9^‐Cnt)] (**1c**) relative to the separated starting materials. Refer to the main text and Supporting Information Chapter 8 for details.

To evaluate the feasibility of option (3), a reaction path optimization with a chain‐of‐states method making rigorous use of a quadratic potential energy surface (TURBOMOLE tool woelfling)^[^ ^65^ ^]^ was performed for Ln = Yb at the PBE0/def2‐SV(P) level (refer to Supporting Information Chapter 8 for details). The result is shown in Figure 5.

In summary, the reaction proceeds via several local minima and transition states, which are all lower in energy than the separated starting materials (refer to Figure 5 and Path.mp4). Five local minima and transition states on the potential energy surface were found. All barriers along the identified reaction path are significantly lower than those calculated for the alternative pathways (1) and (2) outlined above, rendering this reaction pathway the most plausible.

In detail, starting from [Yb^II^(*η* ^9^‐Cnt)_2_] and [Yb^II^(*η* ^8^‐Cot^TIPS^)], a barrierless side‐on coordination of [Yb^II^(*η* ^8^‐Cot^TIPS^)] to [Yb^II^(*η* ^9^‐Cnt)_2_] occurs, resulting in the minimum **M‐I**. The second local minimum (**M‐II**) corresponds to a structure with one tilted Cnt ring and the partially inserted [Yb^II^(*η* ^8^‐Cot^TIPS^)]. Between these two local minima on the potential energy surface, a defined transition state was identified. The imaginary frequency corresponds to an opening of the sandwich structure by one of the Cnt ligands of the [Yb^II^(*η* ^9^‐Cnt)_2_] unit, accompanied by the insertion of the [Yb^II^(*η* ^8^‐Cot^TIPS^)] fragment (refer to Vib_TS‐I.mp4). The barrier height between **M‐I** and **M‐II** amounts to 15 kJ mol^−1^ with respect to **M‐I**.

The transition state between the second (**M‐II**) and the third minimum (**M‐III**) could not be unambiguously identified due to the complexity of the potential energy surface. However, a state with two imaginary frequency vibrations was found, both of which correspond to the further insertion of the [Yb^II^(*η* ^8^‐Cot^TIPS^)] moiety into the opened [Yb^II^(*η* ^9^‐Cnt)_2_] fragment, accompanied by the rotation of one of the *^i^* Pr groups of one TIPS group (refer to Vibs_TS‐II.mp4).

The fourth local minimum (**M‐IV**) already resembles the structure of the final product [(*η* ^9^‐Cnt)Yb^II^(*μ*‐*η* ^8^ *:η* ^8^‐Cot^TIPS^)Yb^II^(*η* ^9^‐Cnt)] (**1c**); however, both TIPS groups are rotated by 120° around the axis connecting the Si atom to the Cot‐C atom it is attached to, resulting in a decreased Ct_Cnt_─Ct_Cot_─Ct_Cnt_ angle compared to the final structure. The localization of a well‐defined transition state between the local minima **M‐III** and **M‐IV** was also unsuccessful. However, again, a state with two imaginary frequency vibrations could be obtained. The highest imaginary frequency vibration again corresponds to the [Yb^II^(*η* ^8^‐Cot^TIPS^)] insertion relevant to the reaction progress, while the second vibration corresponds to the rotation of one of the TIPS groups (refer to Vibs_TS‐III.mp4).

Starting from **M‐IV**, rotation of one of the TIPS groups by 120° results in the minimum **M‐V** which goes over into the final product upon rotation of the second TIPS group by 120°. Between **M‐IV** and **M‐V** and **M‐V** and the final product, respectively, well‐defined transition states with the imaginary frequency corresponding to the respective TIPS rotation and slight changes in the Ct_Cnt_─Ct_Cot_─Ct_Cnt_ angle were identified (refer to Vib_TS‐IV.mp4 and Vib_TS‐V.mp4). The barrier heights along the reaction coordinate amount to 24 and 28 kJ mol^−1^, respectively.

We note that the actual formation of the triple‐decker complexes is likely to follow a more complex mechanism, in which solvent molecules or higher nuclearity aggregates of the starting materials may play a critical role. Nonetheless, for all conceivable alternative reaction pathways, the crucial step is the insertion of the [Ln^II^(*η* ^8^‐Cot^TIPS^)] moiety into the [Ln^II^(*η* ^9^‐Cnt)_2_] fragment, for which option (3) describes the most plausible mechanism.

## Conclusion

In summary, we report the synthesis of two fundamentally new types of sandwich complexes and showcase an unprecedented reaction pathway for building up triple‐decker sandwich complexes. The new complexes are:

i) the first cyclononatetraenyl triple‐decker complexes [(*η* ^9^‐Cnt)Ln^II^(*μ*‐*η* ^8^ *:η* ^8^‐Cot^TIPS^)Ln^II^(*η* ^9^‐Cnt)] (Cnt = C_9_H_9_^‐^) (**1a** = Sm, **1b** = Eu, **1c** = Yb), which are classical sandwich complexes featuring large cyclic, aromatic, all‐carbon ligands. Noteworthy, in **1a–c** the two largest, monocyclic, planar, aromatic solely carbon rings (8‐ and 9‐membered rings) established as ligands in coordination chemistry are present in one triple‐decker complex.

ii) the inverse sandwich compounds [Ln^II^I(thf)_2_(*μ*‐*η* ^8^ *:η* ^8^‐Cot^TIPS^)Ln^II^I(thf)_2_] (Cot^TIPS^ = 1,4‐(*^i^* Pr_3_Si)_2_C_8_H_6_^2−^) (**2a** = Sm, **2b** = Eu) and [Yb^II^(BH_4_)(thf)_2_(*μ*‐*η* ^8^ *:η* ^8^‐Cot^TIPS^)Yb^II^(BH_4_)(thf)_2_] (**2c**), holding promising synthetic potential due to the presence of apical (pseudo‐)halide ligands. These examples also represent the first Cot‐based inverse sandwich compounds.

Critically, we report two distinct synthetic routes for the triple‐decker sandwich complexes: (1) the hitherto unknown insertion of a formal half‐sandwich complex into an existing sandwich system, which was investigated in detail by quantum chemical calculations, and (2) the functionalization of the inverse sandwich compounds **2a**–**c** via classical salt metathesis.

## Supporting Information

The authors have cited additional references within the Supporting Information.^[^ ^66^, ^67^, ^68^, ^69^, ^70^, ^71^, ^72^, ^73^, ^74^, ^75^, ^76^ ^]^

All synthetic protocols, spectroscopic data, supplementary figures and tables, and detailed crystallographic information, photoluminescence measurements, quantum chemical calculations can be found in the supporting information. In detail, these include: Materials and Methods, Synthetic procedures, X‐ray Crystallographic Studies, Photoluminescence measurements, NMR Spectra, Raman Spectra, IR Spectra, and Quantum Chemical Calculations.

## Conflict of Interests

The authors declare no conflict of interest.

## Supporting information



Supporting Information

Supplemental Video 1

Supplemental Video 2

Supplemental Video 3

Supplemental Video 4

Supplemental Video 5

Supporting Information

## Data Availability

The data that support the findings of this study are openly available in RADAR4Chem at http://doi.org/10.17616/R31NJNAY, reference number 1022000. Crystallographic data are available via the Cambridge Crystallographic Data Centre (CCDC): No. 2380916–2380921.
