# Supplementary material for: A Novel Synthetic Pathway to Lanthanide Triple‐Decker Complexes: Selective Expansion of a Sandwich Complex by an Insertion Reaction
Source: Angew Chem Int Ed Engl. 2025 Apr 14;64(25):e202503942. doi: 10.1002/anie.202503942 (PMC12171317; doi:10.1002/anie.202503942)
Supplement: Supplementary file 1 — Supporting Information [file ANIE-64-e202503942-s002.pdf]

Supplementary Materials for

New Synthetic Pathway to Lanthanide Triple-Decker Complexes: Selective Expansion of a Sandwich Complex by an Insertion Reaction

Content

1 Methods S2
2 Synthetic procedures S3
3 X-ray Crystallographic Studies S6
4 Photoluminescence measurements S16
5 NMR Spectra S17
6 Raman Spectra S23
7 IR Spectra S26
8 Quantum Chemical Calculations S28
9 References S33

1 Methods

All manipulations were performed under rigorous exclusion of oxygen and moisture in flame-dried Schlenk-type glassware either on a dual manifold Schlenk line, interfaced to a high vacuum (10^{-3} mbar) pump, or in an argon-filled *MBraun* glove box. Hydrocarbon solvents were pre-dried using a *MBraun* solvent purification system (*SPS-800*), degassed and stored *in vacuo* over $LiAlH_4$. Tetrahydrofuran was additionally distilled under nitrogen over potassium before storage *in vacuo* over $LiAlH_4$. THF-d_8 was dried over Na-K alloy and degassed by freeze-pump-thaw cycles.
Raman spectra were recorded in the region of 4000-20 cm^{-1} on a *Bruker MultiRam* spectrometer equipped with a Nd:YAG laser (λ = 1064 nm) and a germanium detector. Samples were measured in flame sealed glass tubes or glass tubes equipped with a J. Young valve. The laser energy was adjusted to values between 20 and 500 mW depending on the FID amplitude and laser focusing. In terms of their intensity, the signals were classified into different categories (vs = very strong, s = strong, m = medium, w = weak).
NMR spectra were recorded on *Bruker* spectrometers (*Avance II* 300 MHz, *Avance* Neo 400 MHz, or *Avance III* 400 MHz). Chemical shifts are referenced internally using signals of the residual protio solvent (^{1}H) or the solvent ($^{13}C\{^{1}H\}$) and are reported relative to tetramethylsilane (^{1}H, $^{13}C\{^{1}H\}$), or externally relative to tetramethylsilane (^{29}Si), $BF_3{\cdot}Et_2O$ in $CDCl_3$ (^{11}B), or $[Yb(Cp^*)_2(thf)_2]$ in THF (^{171}Yb). All NMR spectra were measured at 298 K unless otherwise stated. The multiplicity of the signals is indicated as s = singlet, d = dublet, q = quartet, qui = quintet, hept = heptet or br s = broad singlet. Assignments were determined based on chemical shifts and integrals where feasible.
Photoluminescence measurements were recorded on a *PTI QuantaMaster™* 8075-22 fluorometer with each double excitation and emission monochromators (*HORIBA Jobin Yvon GmbH*). Emission was detected on a *R928* photomultiplier (250–800 nm) (*HORIBA Jobin Yvon GmbH*). Spectra were corrected for the wavelength-dependent response of the detector and the spectrometer. For detection of the emission decay traces, the sample was excited with either a *Delta Diode™* (*HORIBA Jobin Yvon GmbH*, Model DD-370, λ_{exc} = 371 nm, pulse <2 ns, 2 µW) or a *PTI XenonFLashTM* (set before the emission monochromators, frequency max. 300 MHz). In case of using the *Delta Diode*, the signal was recorded until a satisfying noise to signal ratio was obtained. When using the Xenon Flash lamp, 10'000 traces were recorded. For determination of the lifetimes, the obtained traces were fit with an exponential decay curve (one or two exponentials) using *Origin(Pro)*, Version 2019 (OriginLab Corporation, Northampton, MA, US).
Note: Lifetimes of ≤ 2 ns cannot be determined due to technical limitations of the experimental setup. Hence decay times close to this value are likely to be < 2 ns. Due to the maximum frequency of the used Xenon Flash lamp, a similar limitation applies and decay times determined in the single digit microsecond range (±6 µs) are likely to be shorter than obtained. However, detection of lifetimes in the single digit microsecond area using the *Delta Diode* is often not possible owing to insufficient signal intensity and/or inept excitation wavelength (fixed at the *Delta Diode*). Moreover, as the sample is not fixed and moved for the temperature change, no quantitative comparison of measurements is possible.

[K(Cnt)][1] ,$[K_2(Cot^{TIPS})]$[2] and $[Ln^{II}(Cnt)_2]$[3] were prepared according to literature procedures. All other chemicals were obtained from commercial sources and used without further purification.
Note: To ensure the best possible purity and reliability of all compounds, only crystalline material was isolated. Hence all yields refer to isolated crystalline samples and are generally lower compared to bulk samples.

2 Synthetic procedures

[(η^9-Cnt)Ln^{II}(μ-η^8:η^8-Cot^{TIPS})Ln^{II}(η^9-Cnt)]·(Tol)$_{0.5}$ (1a = Sm, 1b = Eu, 1c = Yb)

Route 1: Toluene (20 mL) was added to a mixture of [Ln^{II}(Cnt)$_2$] (0.160 mmol, 1.00 eq., Ln = Sm: 61.6 mg, Eu: 61.8 mg, Yb: 65.2 mg) and 1/n [Ln^{II}(η^8-Cot^{TIPS})]$_n$ (0.160 mmol, 1.00 eq., Ln = Sm: 90.8 mg, Eu: 91.0 mg, Yb: 94.4 mg). The resulting solution was refluxed for 12 h and then filtered again to remove any insoluble residues. Crystals suitable for X-ray diffraction analysis were obtained by slow evaporation of the toluene solutions at room temperature and subsequent storage at -10°C.

Route 2: To a mixture of the respective anti-bimetallic complex [Ln^{II}I(thf)$_2$(μ-η^8:η^8-Cot^{TIPS})Ln^{II}I(thf)$_2$] (**2a** = Eu, **2b** = Sm) and [Yb^{II}(BH$_4$)(thf)$_2$(μ-η^8:η^8-Cot^{TIPS})Yb^{II}(BH$_4$)(thf)$_2$] (**2c**) (Sm: 184 mg, Eu: 185 mg, Yb: 161 mg, 0.16 mmol, 1.00 eq) and 50 mg [K(Cnt)] (0.32 mmol, 2.00 eq) are added 25 mL of toluene. The reaction mixture is subsequently heated for 12 hours under reflux, and the slow formation of a colorless solid is observed, while no significant change in color occurs. The still warm suspension is then filtered using a PTFE filter. The filtrate is afterwards stored at room temperature for twelve hours, during which time the formation of a single crystalline material occurs (Sm: deep red, Eu: orange, Yb: yellow). If this is not the case, the mother liquor is slowly concentrated under reduced pressure and recrystallized again from hot toluene. Before isolation, the crystallization is completed by storage at -30 °C for two days. After decanting the mother liquor and drying under vacuum, the target compounds can be isolated in the form of crystalline solids. By further concentrating the mother liquor and storing at -30 °C, another batch of crystalline material can be obtained to increase the yield.

Note: According to the ^{1}H NMR spectrum of the diamagnetic complex **1c**, the residual content of non-coordinating toluene found in the molecular structures of compounds **1c** in the solid-state amounts to about half a molecule per complex unit. The elemental analyses below are calculated on the basis of this finding.

1a: Yield Route 1: 45 % (69 mg, 0.072 mmol). **Yield Route 2:** 35 % (56 mg, 0.056 mmol). Calc. for [$C_{47.5}H_{70}Si_2Sm_2$] ([Sm$_2$(Cnt)$_2$(Cot^{TIPS})]·(C_7H_8)$_{0.5}$, 997.97 g·mol^{-1}): C 57.17, H 7.07, found: C 56.51, H 7.37. Due to the paramagnetic character of **1a**, the signal assignment in the ^{1}H NMR spectrum is done under reservation. Integration gives only rough results. No resonances were found in ^{13}C{^{1}H} and ^{29}Si NMR spectra. **^{1}H NMR** (400.3 MHz, THF-d_8): δ [ppm] = 49.82 (br s, C*H*-Cot, FWHM = 2.12 Hz), 44.89 (br s, C*H*-Cot FWHM = 1.82 Hz), 18.48 (s, C*H*-Cnt, FWHM = 14.02 Hz), 7.21-7.07 (m, C*H*-Tol), 2.31 (s, CH_3-Tol), 2.11 (br s, Si(C*H*(CH$_3$)$_2$)$_3$, FWHM = 28.75 Hz), 1.58 (br d, Si(CH(CH_3)$_2$)$_3$). **Raman**: thermal decomposition. **IR (ATR)** $\tilde{\nu}$ [cm^{-1}] = 3083 (w), 3030 (w), 2937 (vs), 2859 (vs), 2719 (w), 1953 (w), 1916 (w), 1816 (w), 1527 (w), 1495 (w), 1458 (s), 1380 (w), 1287 (w), 1243 (w), 1203 (w), 1181 (w), 1068 (w), 1039 (m), 1003 (w), 955 (w), 926 (m), 881 (m), 846 (w), 825 (w), 807 (w), 729 (m), 699 (m), 647 (vs), 600 (m), 518 (w), 495 (w), 466 (w), 450 (w), 421 (w).

1b: Yield Route 1: 46 % (71 mg, 0.074 mmol). **Yield Route 2:** 38 % (61 mg, 0.06 mmol). Calc. for [$C_{47.5}H_{70}Eu_2Si_2$] ([Eu$_2$(Cnt)$_2$(Cot^{TIPS})]·(C_7H_8)$_{0.5}$, 1001.18 g·mol^{-1}): C 56.98, H 7.05, found: C 56.36, H 6.90. Due to the paramagnetic nature of **1b**, no meaningful NMR spectra of the compound could be obtained. **Raman**: $\tilde{\nu}$ [cm^{-1}] = 3051 (w), 3009 (w), 2913 (w), 2863 (m), 1604 (w), 1521 (w), 1481 (w), 1444 (w), 1290 (w), 1238 (w), 1209 (w), 1157 (w), 1067 (w), 1029 (w), 1005 (w), 972 (w), 882 (w), 786 (w), 752 (w), 704 (w), 675 (vs), 592 (w), 523 (w), 479 (w), 426 (w), 337 (w), 278 (s), 220 (w), 123 (vs).

1c: Yield Route 1: 41 % (66 mg, 0.066 mmol). **Yield Route 2:** 32 % (53 mg, 0.51 mmol). Calc. for [$C_{47.5}H_{70}Si_2Yb_2$] ([Yb$_2$(Cnt)$_2$(Cot^{TIPS})]·(C_7H_8)$_{0.5}$, 1043.36 g·mol^{-1}): C 54.68, H 6.76, found: C 54.12, H 7.07.

Due to low signal intensity, not all resonances of the non-coordinating toluene molecules are found in the $^{13}C\{^{1}H\}$ NMR spectrum of **1c**. **^{1}H NMR** (400.3 MHz, THF-d_8): δ [ppm] = 7.20-7.06 (m, 2.5 H, C*H*-Tol), 6.94 (br s, 18 H, C*H*-Cnt), 6.28 (m, 2 H, C*H*-Cot), 6.23-6.17 (m, 2 H, C*H*-Cot), 6.09-6.03 (m, 2 H, C*H*-Cot), 1.53 (s, 1.5 H, CH_3-Tol), 1.54 (hept, $^{3}J_{HH}$ = 7.5 Hz, 6 H, Si(C*H*(CH_3)$_2$)$_3$), 1.16 (d, $^{3}J_{HH}$ = 7.4 Hz, 18 H, Si(CH(CH_3)$_2$)$_3$), 1.14 (d, $^{3}J_{HH}$ = 7.4 Hz, 18 H, Si(CH(CH_3)$_2$)$_3$). **^{13}C{^{1}H} NMR** (75.48 MHz, THF-d_8): δ [ppm] = 129.8 (*C*-Tol), 129.1 (*C*-Tol), 126.2 (*C*-Tol), 109.2 (*C*-Cnt), 99.2 (*C*-Cot), 98.9 (*C*-Cot), 93.7 (*C*-Cot), 87.5 (*C*-Cot), 20.5 (Si(CH(*C*H$_3$)$_2$)$_3$), 20.4 (Si(CH(*C*H$_3$)$_2$)$_3$), 14.0 (Si(*C*H(CH$_3$)$_2$)$_3$). **^{29}Si{^{1}H} NMR** (59.63 MHz, THF-d_8): δ [ppm] = 5.1 (*Si*^{i}Pr$_3$). **^{171}Yb NMR** (52.54, THF-d_8): δ [ppm] = 492.0. **Raman**: $\tilde{\nu}$ [cm^{-1}] = 3049 (w), 3010 (w), 2942 (w), 2912 (w), 2866 (w), 2704 (w), 1521 (w), 1475 (w), 1386 (w), 1362 (w), 1287 (w), 1250 (w), 1159 (w), 1068 (w), 1016 (w), 973 (w), 945 (w), 883 (w), 753 (w), 682 (s), 660 (w), 569 (w), 521 (w), 473 (w), 439 (w), 280 (m), 106 (vs).

[Ln^{II}I(thf)$_2$(μ-η^8:η^8-Cot^{TIPS})Ln^{II}I(thf)$_2$] (2a = Sm, 2b = Eu) and [Yb^{II}(BH$_4$)(thf)$_2$(μ-η^8:η^8-Cot^{TIPS})Yb^{II}(BH$_4$)(thf)$_2$] (2c)

To a mixture of [Ln^{II}I$_2$(thf)$_2$] (for **2a** and **2b**) (Sm: 1108 mg, Eu: 1110 mg, 2.02 mmol, 2.00 eq) or [Yb^{III}(BH$_4$)$_3$(thf)$_3$] (for **2c**) (446 mg, 1.01 mmol, 1.00 eq) and 500 mg [K$_2$(Cot^{TIPS})] (1.01 mmol, 1.00 eq) are added 25 mL THF at -78 °C. The reaction mixture is then warmed to room temperature and stirred for twelve hours, during which time a gradual yellow (Eu, Yb) or red (Sm) coloration of the mixture is observed, respectively. Then, the reaction solvent is removed under reduced pressure and the remaining residue is extracted with hot toluene and filtered through a glass frit until the extract remains colorless. The filtrate is subsequently concentrated until the clear formation of a yellow (Eu, Yb) or red (Sm) solid is observable. Then THF is added until the complete precipitate can be dissolved in a hot solution (approximate volume ratio: 5:1 (toluene:THF)). If small amounts of insoluble solid remain, it can be removed by filtration through a PTFE filter. Single crystals of compounds **2a-c** suitable for X-ray structural analysis can be obtained by storing the hot saturated toluene/THF mixtures at room temperature. Finally, after storage at -30 °C for two days, decantation of the mother liquor and drying under vacuum, the products are isolated as yellow (Eu, Yb) and red (Sm) crystalline solids, respectively.

2a: Yield: 39 % (454 mg, 0.39 mmol). Calc. for [$C_{36}H_{68}I_2O_{2.5}Si_2Sm_2$] ([Sm$_2I_2$(Cot^{TIPS})(thf)$_{2.5}$], 1151.64 g·mol^{-1}): C 37.55, H 5.95, found: C 37.18, H 5.33. Due to the paramagnetic character of **2a**, the signal assignment and integration of the ^{1}H NMR spectrum is uncertain. In addition, the proton spectrum reported here was recorded at 203 K, since only very broad and not well-defined signals were found at 298 K. No resonances were observed in ^{13}C{^{1}H}- and ^{29}Si-NMR spectra. Due to signal overlaps of the THF-h_8 and THF-d_7h_1 signals in the proton spectrum, no reliable integration can be performed; accordingly, the exact residual THF content of the isolated compound can only be estimated on the basis of elemental analysis. For this purpose, the assumption of 2.5 molecules of THF per complex unit is made. **^{1}H NMR** (400.3 MHz, 203 K, THF-d_8): δ [ppm] = 69.46 (s, 2 H, C*H*-Cot, FWHM = 116.45 Hz), 55.15 (s, 2 H, C*H*-Cot, FWHM = 97.31 Hz), 20.22 (s, 2 H, C*H*-Cot, FWHM = 68.93 Hz), 2.77 and 2.52 (two s, 36 H, Si(CH(CH_3)$_2$)$_3$), 1.53 (s, 6 H, Si(C*H*(CH$_3$)$_2$)$_3$, FWHM = 27.66 Hz). **Raman**: thermal decomposition. **IR (ATR)**: $\tilde{\nu}$ [cm^{-1}] = 2941 (s), 2888 (m), 2862 (vs), 1459 (w), 1381 (w), 1365 (w), 1342 (w), 1240 (w), 1198 (w), 1069 (w), 1030 (s), 996 (w), 924 (m), 878 (m), 837 (w), 777 (w), 741 (m), 667 (w), 655 (w), 636 (m), 608 (w), 595 (w), 577 (w), 520 (w).

2b: Yield: 54 % (630 mg, 0.55 mmol). Analogous to **2a**, the elemental analysis is calculated by assuming a residual content of 2.5 THF molecules per complex unit. Calc. for [$C_{36}H_{68}Eu_2I_2O_{2.5}Si_2$] ([Eu$_2I_2$(Cot^{TIPS})(thf)$_{2.5}$], 1154.84 g·mol^{-1}): C 37.44, H 5.94, found: C 37.23, H 6.08. Due to the

paramagnetic nature of **2b**, no meaningful NMR spectra of the compound could be obtained. **Raman**: $\tilde{\nu}$ [cm^{-1}] = 3040 (w), 2969 (m), 2940 (m), 2890 (m), 2864 (vs), 2756 (w), 2711 (w), 2579 (w), 1529 (w), 1479 (m), 1366 (w), 1344 (w), 1295 (w), 1236 (w), 1157 (w), 1070 (w), 1033 (w), 971 (w), 921 (w), 881 (m), 779 (w), 750 (m), 666 (w), 637 (w), 584 (w), 519 (w), 486 (w), 435 (w), 355 (w), 329 (w), 282 (w), 201 (w). **IR (ATR)**: decomposition.

2c: Yield: 44 % (448 mg, 0.44 mmol). Both, the ^{1}H NMR spectrum and the elemental analysis indicate a residual content of three THF molecules per complex unit. Calc. for [$C_{38}H_{80}B_2O_3Si_2Yb_2$] ([$Yb_2I_2(Cot^{TIPS})(thf)_3$], 1008.95 g·$mol^{-1}$): C 45.24, H 7.99, found: C 45.33, H 7.67. **^{1}H NMR** (300.13 MHz, THF-d_8): δ [ppm] = 6.31 (m, 2 H, C*H*-Cot), 6.27-6.18 (m, 2 H, C*H*-Cot), 6.12-6.04 (m, 2 H, C*H*-Cot), 3.64-3.59 (m, 12 H, CH_2-thf), 1.79-1.75 (m, 12 H, CH_2-thf), 1.56 (hept, $^{3}J_{HH}$ = 7.4 Hz, 6 H, Si(C*H*(CH_3)$_2$)$_3$), 1.15 (d, $^{3}J_{HH}$ = 7.4 Hz, 36 H, Si(CH(CH_3)$_2$)$_3$), 0.88-0.06 (br q, $^{1}J_{HB}$ = 83 Hz, 8 H, BH_4). **^{11}B NMR** (96.29 MHz, THF-d_8): δ [ppm] = -34.6 (qui, $^{1}J_{HB}$ = 82 Hz, *B*H_4). **^{13}C{^{1}H} NMR** (75.48 MHz, THF-d_8): δ [ppm] = 97.9 (*C*-Cot), 97.8 (*C*-Cot), 92.5 (*C*-Cot), 86.3 (*C*-Cot), 67.2 (*C*-thf), 25.4 (*C*-thf), 19.3 (Si(CH(*C*H_3)$_2$)$_3$), 12.83 (Si(*C*H(CH_3)$_2$)$_3$). **^{29}Si{^{1}H} NMR** (59.63 MHz, THF-d_8): δ [ppm] = 5.2 (*Si*^{i}Pr$_3$). **^{171}Yb{^{1}H} NMR** (52.53 MHz, THF-d_8): δ [ppm] = 338.7, 310.5. **Raman**: $\tilde{\nu}$ [cm^{-1}] = 3047 (w), 2943 (m), 2864 (vs), 2756 (w), 2708 (w), 2386 (w), 2267 (w), 2159 (w), 1481 (m), 1448 (m), 1382 (w), 1367 (w), 1295 (w), 1235 (w), 1158 (w), 1072 (w), 1036 (w), 995 (w), 970 (w), 923 (w), 882 (m), 753 (s), 668 (w), 638 (w), 569 (w), 519 (w), 488 (w), 440 (w), 353 (w), 282 (w), 195 (w). **IR (ATR)**: decomposition.

3 X-ray Crystallographic Studies

Suitable crystals were selected under an optic microscope equipped with polarizing filters, covered in mineral oil (Aldrich) and mounted on a *MiTeGen* holder. The crystals were transferred directly to the cold stream of a STOE IPDS 2 or *STOE StadiVari* diffractometer, equipped with a Mo-sealed tube, a *MoGenix 3D HF* or *Ga-Metaljet* X-ray source (Ga-K_α = 1.34012 Å, Mo-K_α = 0.71073 Å).

All structures were solved using the programs *SHELXS/T* and *Olex2 1.2*.[4],[5],[6] The remaining non-hydrogen atoms were located from successive difference Fourier map calculations. The refinements were carried out by using full-matrix least-squares techniques on F^2 by using the program *SHELXL*. In each case, the locations of the largest peaks in the final difference Fourier map calculations, as well as the magnitude of the residual electron densities, were of no chemical significance.

Specific comments on the structures discussed here are given in the following section.

1a [(η^9-Cnt)Sm^{II}(μ-η^8:η^8-Cot^{TIPS})Sm^{II}(η^9-Cnt)]·$(Tol)_{0.5}$: The molecular structure of **1a** exhibits Cnt ligands that are disordered in a 70:30 ratio across a pseudo ecliptic and a pseudo staggered conformation. Refinement of the Cnt system was performed with the aid of moderate RIGU restraints. In addition, the statistically, over three positions, disordered TIPS groups were modeled using SIMU restraints. Furthermore, a half occupied non-coordinating molecule of toluene in the asymmetric unit of **1a** could not be modeled satisfactorily. Therefore, the correlated electron density was removed from the electron density map of the asymmetric unit with the *solvent mask algorithm* of *Olex2 1.5*.[6]

1b [(η^9-Cnt)Eu^{II}(μ-η^8:η^8-Cot^{TIPS})Eu^{II}(η^9-Cnt)]·$(Tol)_{0.5}$: The molecular structure of **1a** exhibits Cnt ligands that are disordered in a 70:30 ratio across a pseudo ecliptic and a pseudo staggered conformation. Refinement of the Cnt system was performed with the aid of SIMU restraints. In addition, the statistically, over three positions, disordered TIPS groups were modeled using SIMU and SADI restraints. Close inspection of the residual electron density above the Cnt system suggests the presence of another disordered *cis-cis-cis-trans* Cnt unit, which, however, could not be stably refined due to the low chemical population. Furthermore, a half occupied non-coordinating molecule of toluene in the asymmetric unit of **1b** could not be satisfactorily modeled, so the correlated electron density was removed from the electron density map of the asymmetric unit with the *solvent mask algorithm* of *Olex2 1.5*.[6]

1c [(η^9-Cnt)Yb^{II}(μ-η^8:η^8-Cot^{TIPS})Yb^{II}(η^9-Cnt)]·$(Tol)_{0.5}$: The Cnt ligands were refined using light RIGU constraints. In the case of the TIPS groups, SIMU and SADI constraints were used. Furthermore, a half occupied non-coordinating molecule of toluene in the asymmetric unit of **1c** could not be satisfactorily modeled, so the correlated electron density was removed from the electron density map of the asymmetric unit with the *solvent mask algorithm* of *Olex2 1.5*.[6]

PLAT972_ALERT_2_B Check Calcd Resid. Dens. Yb01

Response: Fourier truncation error.

2a [$Sm^{II}I(thf)_2$(μ-η^8:η^8-Cot^{TIPS})$Sm^{II}I(thf)_2$]: The molecular solid state structure of compound **2a** features disordered isopropyl groups and THF ligands. Both were modelled using light to moderate SIMU and SADI restraints.

2b [$Eu^{II}I(thf)_2(\mu\text{-}\eta^8\text{:}\eta^8\text{-}Cot^{TIPS})Eu^{II}I(thf)_2$]: The molecular solid state structure of compound **2b** features disordered isopropyl groups and THF ligands. Both were modelled using moderate SIMU restraints.

2c [$Yb^{II}(BH_4)(thf)_2(\mu\text{-}\eta^8\text{:}\eta^8\text{-}Cot^{TIPS})Yb^{II}(BH_4)(thf)_2$]: The solution was performed completely half-filled in *P*-1, transferring the entire molecule to part -1. There is an inversion center in the middle of the molecule/C_8 ring, whereby the missing half is generated inverted (grow). Otherwise, a correct structural solution was not possible without going to *P*1 (which, however, means no information gain). For the disordered position at C32a/b and C37a/b, part -2 was used, which prevented a correct calculation of the hydrogen atoms. In order to obtain the correct sum formula, the hydrogen atoms in C32a and C37a were therefore not weighted like the disordered part, but 0.5.

Crystallographic data for the structures reported in this paper have been deposited with the Cambridge Crystallographic Data Centre as a supplementary publication no. 2380916-2380921. Copies of the data can be obtained free of charge on application to CCDC, 12 Union Road, Cambridge CB21EZ, UK (fax: (+(44)1223-336-033; email: deposit@ccdc.cam.ac.uk).

Table S1: Crystal data and structure refinement for compounds **1a-c**.

Compound	**1a**	**1b**	**1c**
Formula	$C_{51}H_{74}Si_2Sm_2$	$C_{51}H_{74}Eu_2Si_2$	$C_{51}H_{74}Si_2Yb_2$
$\rho_{calc.}$/ g cm^{-3}	1.488	1.493	1.553
μ/mm^{-1}	2.580	2.751	4.073
Formula Weight	1043.98	1047.20	1089.36
Color	red	clear orange	orange
Shape	fragment-shaped	prism-shaped	prism-shaped
Size/mm^3	0.34×0.24×0.19	0.36×0.25×0.07	0.36×0.25×0.07
T/K	100	100	100
Crystal System	orthorhombic	orthorhombic	orthorhombic
Space Group	*Cmcm*	*Cmcm*	*Cmcm*
a/Å	17.173(2)	17.173(2)	17.173(2)
b/Å	15.124(2)	15.124(2)	15.124(2)
c/Å	17.943(2)	17.943(2)	17.943(2)
α/°	-	-	-
β/°	-	-	-
γ/°	-	-	-
V/Å^3	4660.2(8)	4660.2(8)	4660.2(8)
Z	4	4	4
Z'	0.25	0.25	0.25
Wavelength/Å	0.71073	0.71073	0.71073
Radiation type	Mo K_{α}	Mo K_{α}	Mo K_{α}
Θ_{min}/°	2.123	2.123	2.123
Θ_{max}/°	31.210	31.462	32.053
Measured Refl.	13480	13969	11737
Independent Refl.	3594	3535	3475
Reflections with I > 2(I)	2961	2822	2630
R_{int}	0.0275	0.0331	0.0566
Parameters	214	214	124
Restraints	72	42	39
Largest Peak	1.604	1.255	2.880
Deepest Hole	-1.472	-0.665	-3.052
GooF	1.151	1.075	1.057
wR_2 (all data)	0.0791	0.0776	0.1477
wR_2	0.0769	0.0742	0.1379
R_1 (all data)	0.0489	0.0437	0.0929
R_1	0.0382	0.0316	0.0706

Table S2: Crystal data and structure refinement for **2a-c.**

Compound	**2a**	**2b**	**2c**
Formula	$C_{42}H_{80}I_2O_4Si_2Sm_2$	$C_{42}H_{80}Eu_2I_2O_4Si_2$	$C_{42}H_{88}B_2O_4Si_2Yb_2$
$\rho_{calc.}$/ g cm^{-3}	1.698	1.684	1.521
μ/mm^{-1}	3.696	20.402	4.024
Formula Weight	1259.74	1262.96	1081.00
Color	purple	yellow	yellow
Shape	rod-shaped	needle-shaped	fragment-shaped
Size/mm^3	0.44×0.19×0.04	0.83×0.64×0.40	0.33×0.24×0.18
T/K	100	150	100
Crystal System	orthorhombic	orthorhombic	triclinic
Space Group	*Pnma*	*Pnma*	*P*-1
a/Å	25.6251(5)	25.6143(9)	8.4119(3)
b/Å	22.4371(7)	22.6290(6)	13.2994(5)
c/Å	8.5723(2)	8.5920(2)	12.5187(4)
α/°	-	-	115.821(3)
β/°	-	-	89.976(3)
γ/°	-	-	108.441(3)
V/Å^3	4928.7(2)	4980.1(2)	1180.37(8)
Z	4	4	1
Z'	0.5	0.5	0.5
Wavelength/Å	0.71073	1.34143	0.71073
Radiation type	Mo K_α	Ga K_α	Mo K_α
Θ_{min}/°	2.505	3.002	3.634
Θ_{max}/°	29.469	64.233	58.762
Measured Refl.	28306	45677	36701
Independent Refl.	6552	6375	6395
R_{int}	0.0341	0.0671	0.0689
Parameters	333	285	525
Restraints	72	30	192
Largest Peak	0.932	2.631	1.97
Deepest Hole	-0.993	-1.891	-1.66
GooF	1.027	1.008	1.101
wR_2 (all data)	0.0763	0.1594	0.1174
wR_2	0.0697	0.1502	0.1136
R_1 (all data)	0.0598	0.0743	0.0556
R_1	0.0368	0.0562	0.0468

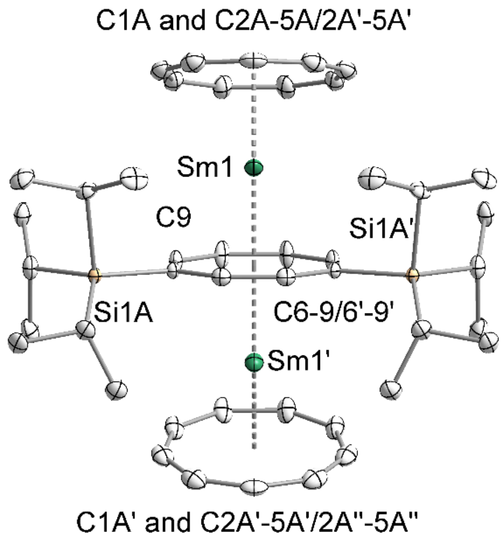

Figure S1: Molecular structure of [(η^9-Cnt)Sm^{II}(μ-η^8:η^8-Cot^{TIPS})Sm^{II}(η^9-Cnt)] (**1a**) in the solid state. Thermal ellipsoids are represented at 50% probability. Hydrogen atoms are omitted for clarity. Only one part of the disordered Cnt ligands and TIPS groups is depicted.

Table S3: Selected bond lengths, distances and angles of [(η^9-Cnt)Sm^{II}(μ-η^8:η^8-Cot^{TIPS})Sm^{II}(η^9-Cnt)] (**1a**). Ct = centroid.

Selected bond lengths and distances [Å]		Selected angles [°]	
Sm1-C$_{Cot}$	2.786(3)-2.896(3)	Sm1-Ct$_{Cot}$-Sm1’	175.849(14)
Sm1-Ct$_{Cot}$	2.1551(4)		
Sm1-Sm1’	4.3073(7)		
Part A			
Sm1-C$_{Cnt}$	2.884(9)-2.945(5)	Ct$_{Cnt}$-Sm1-Ct$_{Cot}$	163.86(2)
Sm1-Ct$_{Cnt}$	2.0949(4)	Ct$_{Cnt}$-Ct$_{Cot}$-Ct-$_{Cnt}$	159.935(2)
Part B			
Sm1-C$_{Cnt}$	2.95(2)-2.985(13)	Ct$_{Cnt}$-Sm1-Ct$_{Cot}$	167.38(2)
Sm1-Ct$_{Cnt}$	2.1605(4)	Ct$_{Cnt}$-Ct$_{Cot}$-Ct-$_{Cnt}$	163.210(2)

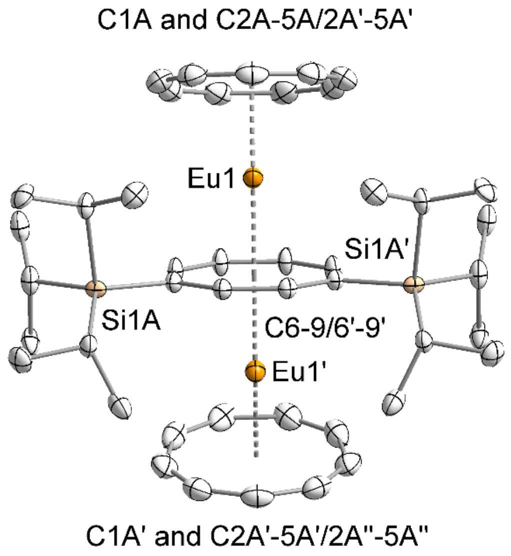

Figure S2: Molecular structure of [(η^9-Cnt)Eu^{II}(μ-η^8:η^8-Cot^{TIPS})Eu^{II}(η^9-Cnt)] (**1b**) in the solid state. Thermal ellipsoids are represented at 50% probability. Hydrogen atoms are omitted for clarity. Only one part of the disordered Cnt ligands and TIPS groups is depicted.

Table S4: Selected bond lengths, distances and angles of [(η^9-Cnt)Eu^{II}(μ-η^8:η^8-Cot^{TIPS})Eu^{II}(η^9-Cnt)] (**1b**). Ct = centroid.

Selected bond lengths and distances [Å]		Selected angles [°]	
Eu1-C_{Cot}	2.786(2)-2.891(2)	Eu1-Ct_{Cot}-Eu1’	175.901(14)
Eu1-Ct_{Cot}	2.1505(4)		
Eu1-Eu1’	4.2983(7)		
Part A			
Eu1-C_{Cnt}	2.900(5)-2.950(4)	Ct_{Cnt}-Eu1-Ct_{Cot}	164.24(2)
Eu1-Ct_{Cnt}	2.1004(4)	Ct_{Cnt}-Ct_{Cot}-Ct-$_{Cnt}$	160.324(2)
Part B			
Eu1-C_{Cnt}	2.849(14)-2.985(14)	Ct_{Cnt}-Eu1-Ct_{Cot}	167.74(2)
Eu1-Ct_{Cnt}	2.1644(4)	Ct_{Cnt}-Ct_{Cot}-Ct-$_{Cnt}$	163.604(2)

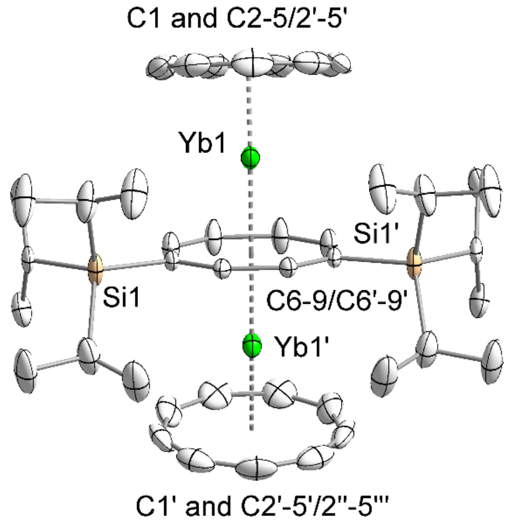

Figure S3: Molecular structure of [(η^9-Cnt)Yb^{II}(μ-η^8:η^8-Cot^{TIPS})Yb^{II}(η^9-Cnt)] (**1c**) in the solid state. Thermal ellipsoids are represented at 50% probability. Hydrogen atoms are omitted for clarity.

Table S5: Selected bond lengths, distances and angles of [(η^9-Cnt)Yb^{II}(μ-η^8:η^8-Cot^{TIPS})Yb^{II}(η^9-Cnt)] (**1c**). Ct = centroid.

Selected bond lengths and distances [Å]		Selected angles [°]	
Yb1-C_{Cnt}	2.833(9)-2.866(10)	Yb1-Ct_{Cot}-Yb1’	175.490(2)
Yb1-C_{Cot}	2.710(7)-2.858(7)	Ct_{Cnt}-Yb1-Ct_{Cot}	169.50(3)
Yb1-Ct_{Cnt}	2.0333(6)	Ct_{Cnt}-Ct_{Cot}-Ct-$_{Cnt}$	165.157(2)
Yb1-Ct_{Cot}	2.1009(6)		
Yb1-Yb1’	4.1986(9)		

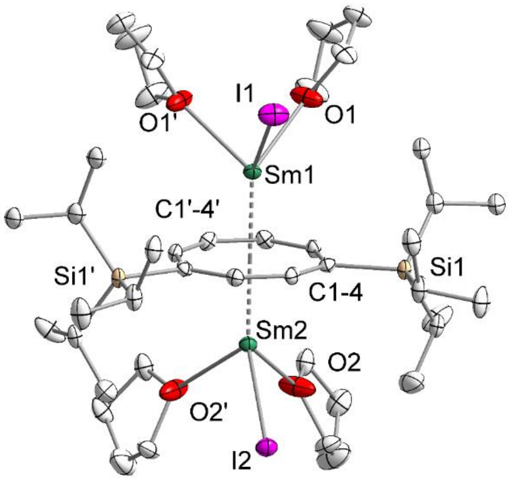

Figure S4: Molecular structure of [Sm^{II}I(thf)$_2$(μ-η^8:η^8-Cot^{TIPS})Sm^{II}I(thf)$_2$] (**2a**) in the solid state. Thermal ellipsoids are represented at 50% probability. Hydrogen atoms are omitted for clarity. Only one part of the statistically disordered THF ligand is depicted.

Table S6: Selected bond lengths, distances and angles of [Sm^{II}I(thf)$_2$(μ-η^8:η^8-Cot^{TIPS})Sm^{II}I(thf)$_2$] (**2a**). Ct = centroid.

Selected bond lengths and distances [Å]		Selected angles [°]	
Sm1-I1	3.1687(5)	Sm1-Ct$_{Cot}$-Sm2	179.81(2)
Sm1-O1	2.541(3)	I1-Sm1-Ct$_{Cot}$	131.12(2)
Sm1-C$_{Cot}$	2.849(4)-2.913(4)	I2-Sm2-Ct$_{Cot}$	126.97(2)
Sm1-Ct$_{Cot}$	2.1885(5)	I1-Sm1-Sm2-I2	0.00(3)
Sm2-I2	3.1837(5)		
Sm2-O2	2.548(3)		
Sm2-C$_{Cot}$	2.824(4)-2.877(4)		
Sm2-Ct$_{Cot}$	2.1578(5)		
Sm1-Sm2	4.3462(7)		

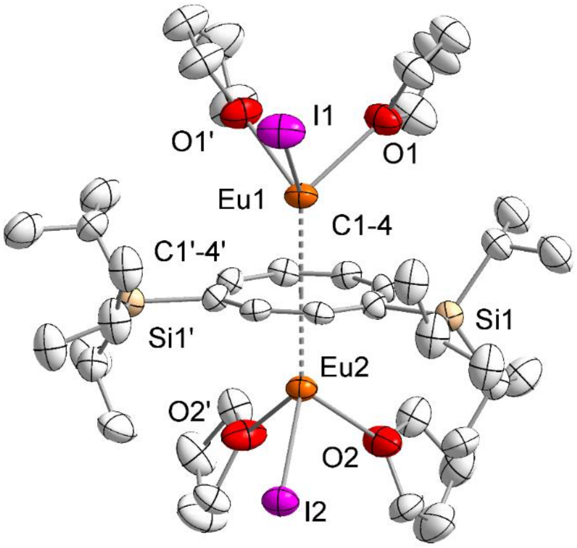

Figure S5: Molecular structure of [Eu^{II}I(thf)$_2$(μ-η^8:η^8-Cot^{TIPS})Eu^{II}I(thf)$_2$] (**2b**) in the solid state. Thermal ellipsoids are represented at 50% probability. Hydrogen atoms are omitted for clarity. Only one part of the statistically disordered isopropyl groups and THF ligands is depicted.

Table S7: Selected bond lengths, distances and angles of [Eu^{II}I(thf)$_2$(μ-η^8:η^8-Cot^{TIPS})Eu^{II}I(thf)$_2$] (**2b**). Ct = centroid.

Selected bond lengths and distances [Å]		Selected angles [°]	
Eu1-I1	3.1505(8)	Eu1-Ct$_{Cot}$-Eu2	179.55(2)
Eu1-O1	2.530(5)	I1-Eu1-Ct$_{Cot}$	130.80(2)
Eu1-C$_{Cot}$	2.844(6)-2.911(6)	I2-Eu2-Ct$_{Cot}$	126.58(2)
Eu1-Ct$_{Cot}$	2.1905(5)	I1-Eu1-Eu2-I2	0.00(3)
Eu2-I2	3.1689(8)		
Eu2-O2	2.538(5)		
Eu2-C$_{Cot}$	2.827(6)-2.8761(59)		
Eu2-Ct$_{Cot}$	2.1626(5)		
Eu1-Eu2	4.3531(7)		

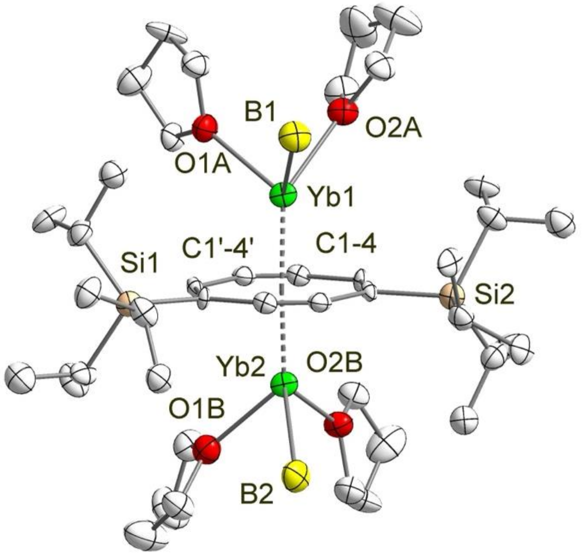

Figure S6: Molecular structure of [Yb^{II}(BH$_4$)(thf)$_2$(μ-η^8:η^8-Cot^{TIPS})Yb^{II}(BH$_4$)(thf)$_2$] (**2c**) in the solid state. Thermal ellipsoids are represented at 50% probability. Hydrogen atoms are omitted for clarity. Only one part of the statistically disordered isopropyl groups and THF ligands is depicted.

Table S8: Selected bond lengths, distances and angles of [Yb^{II}(BH$_4$)(thf)$_2$(μ-η^8:η^8-Cot^{TIPS})Yb^{II}(BH$_4$)(thf)$_2$] (**2c**). Ct = centroid.

Selected bond lengths and distances [Å]		Selected angles [°]	
Yb1-B1	2.764(14)	Yb1-Ct$_{Cot}$-Yb2	167.12(13)
Yb1-O2A	2.449(9)	B1-Yb1-Ct$_{Cot}$	122.556(2)
Yb1-O1A	2.380(9)	B2-Yb2-Ct$_{Cot}$	122.288(2)
Yb2-B2	2.739(14)		
Yb2-O2B	2.344(8)		
Yb2-O1B	2.416(10)		
Yb1-C$_{Cot}$	2.628(15)-2.932(15)		
Yb1-Ct$_{Cot}$	2.086(4)		
Yb1-Yb2	4.1888(4)		

4 Photoluminescence measurements

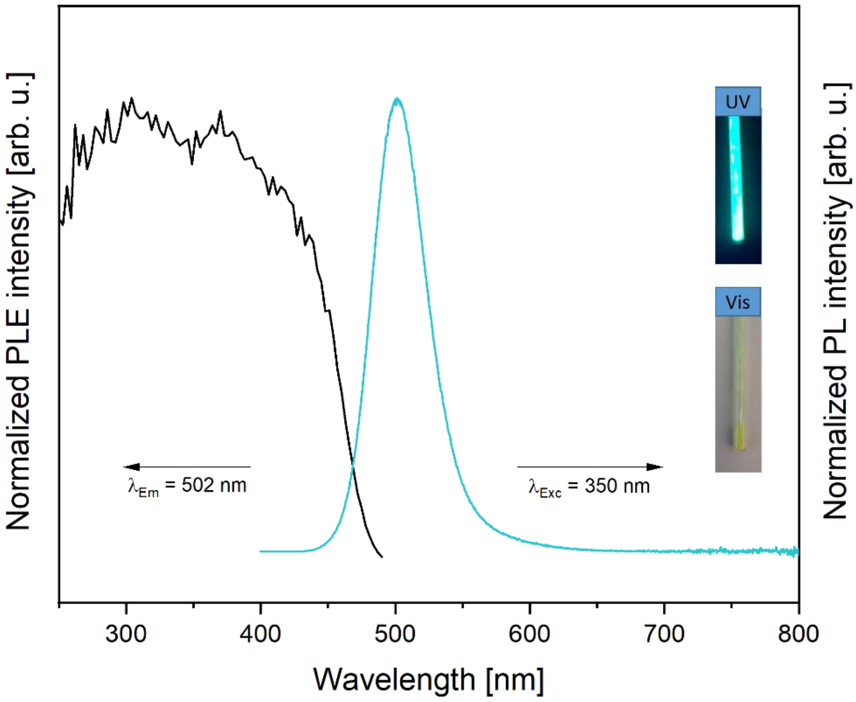

Figure S7: Solid-state photoluminescence emission (PL) and excitation (PLE) spectra of **2b** at room temperature. The PL emission and the PLE spectrum was recorded at the indicated wavelengths and normalized. The pictures on the right show the sample under UV lamp illumination (365 nm) and daylight.

5 NMR Spectra

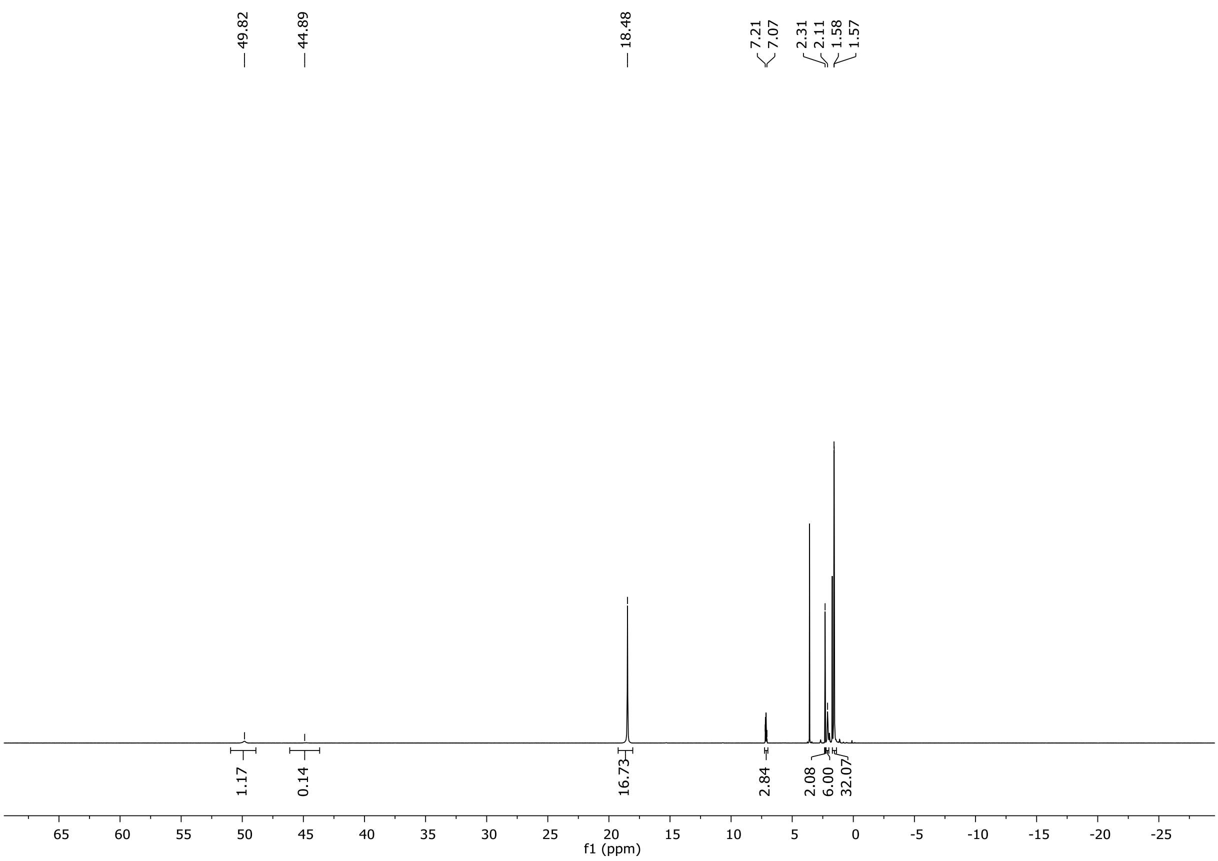

Figure S8: ^{1}H NMR spectrum of **1a** in THF-d_8. Note, compound **1a** is paramagnetic and integration is not reliable.

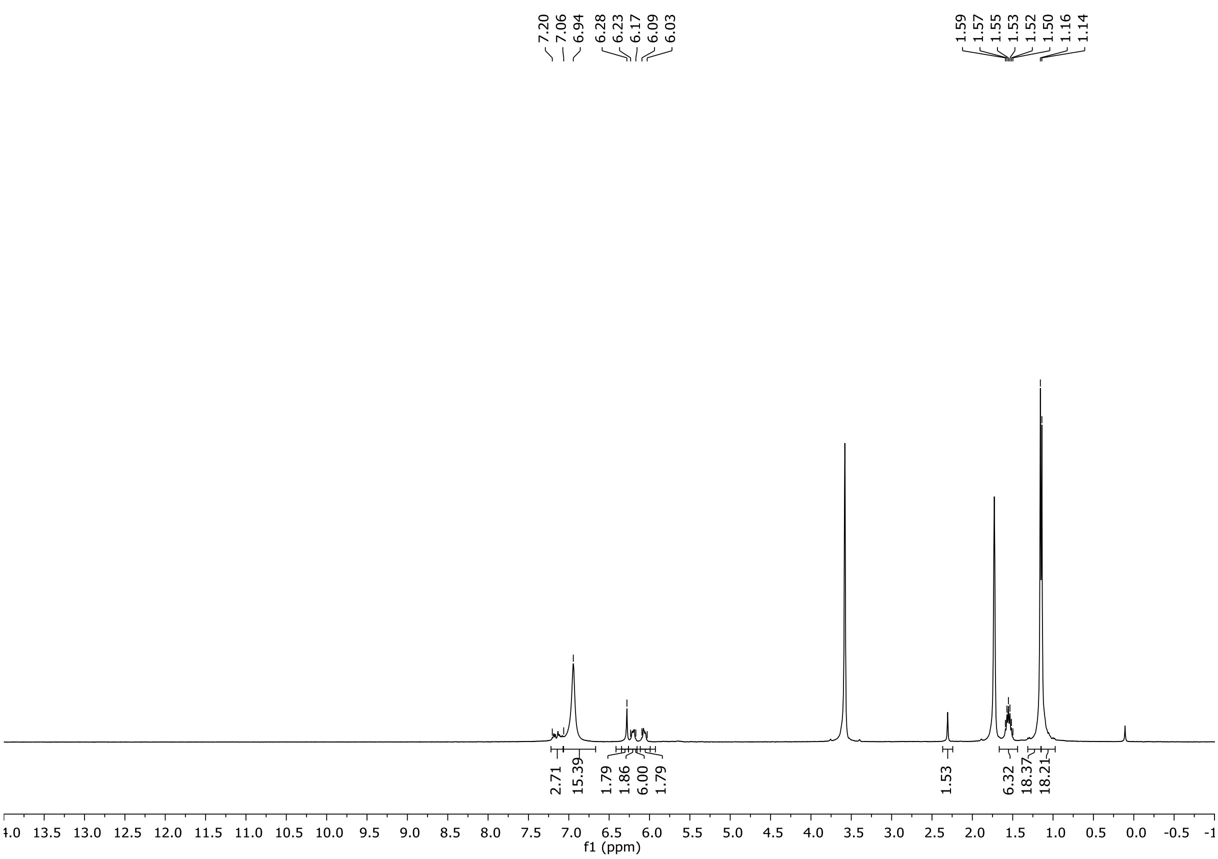

Figure S9: ^{1}H NMR spectrum of **1c** in THF-d_8.

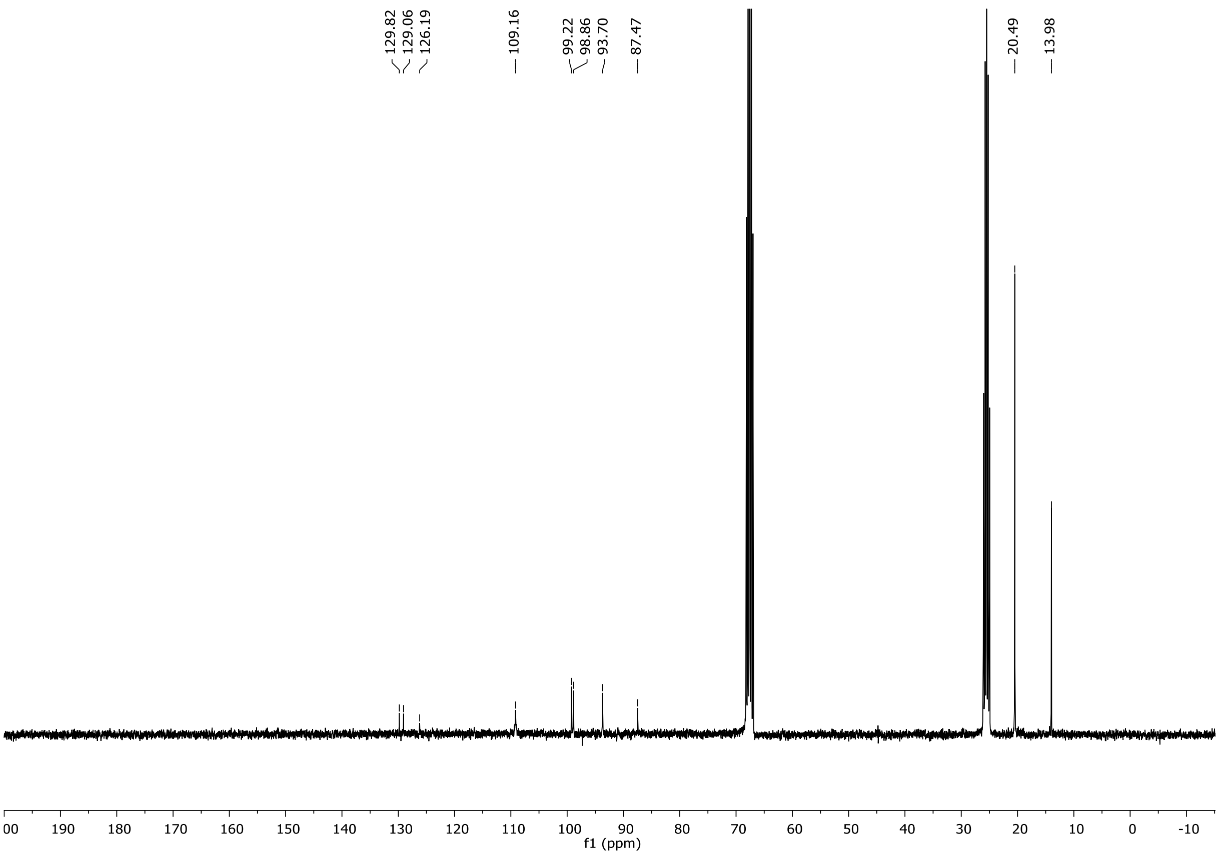

Figure S10: $^{13}C\{^1H\}$ NMR spectrum of **1c** in THF-d_8.

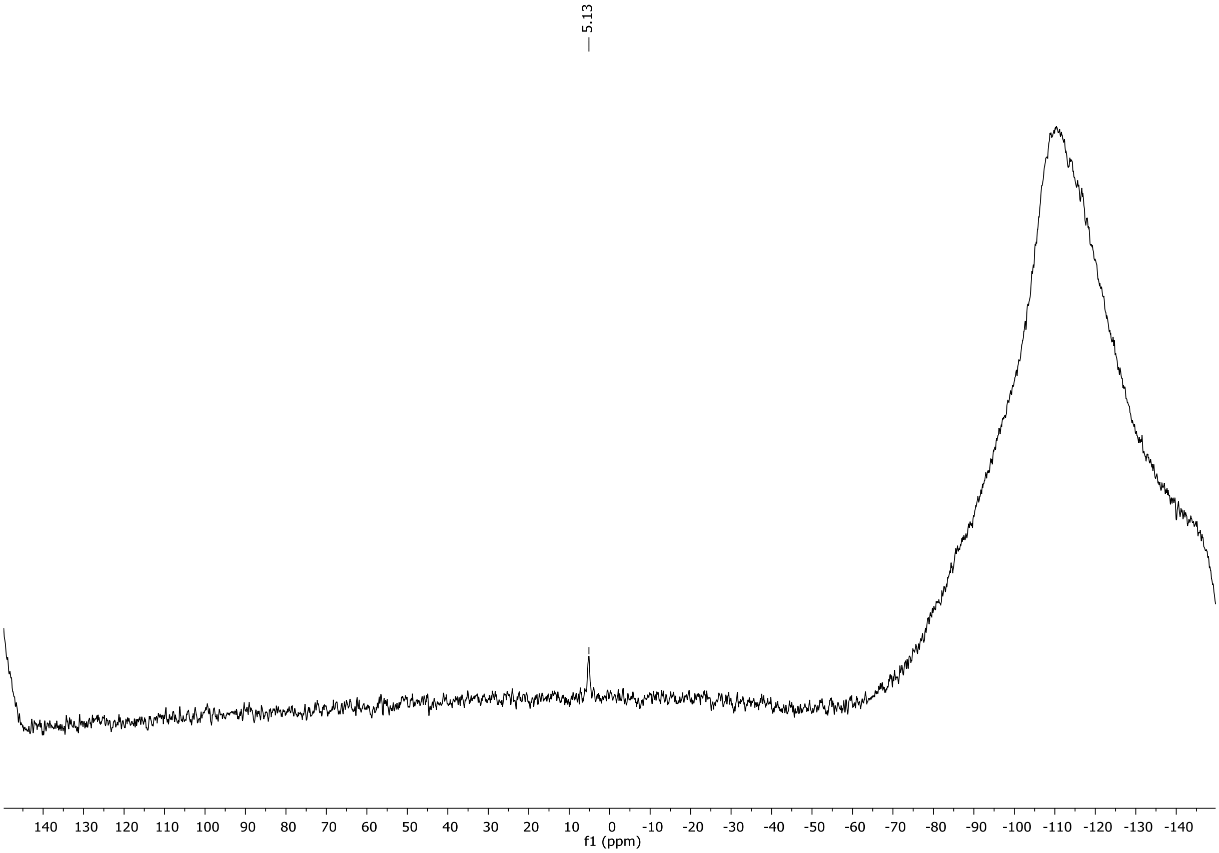

Figure S11: ^{29}Si NMR spectrum of **1c** in THF-d_8.

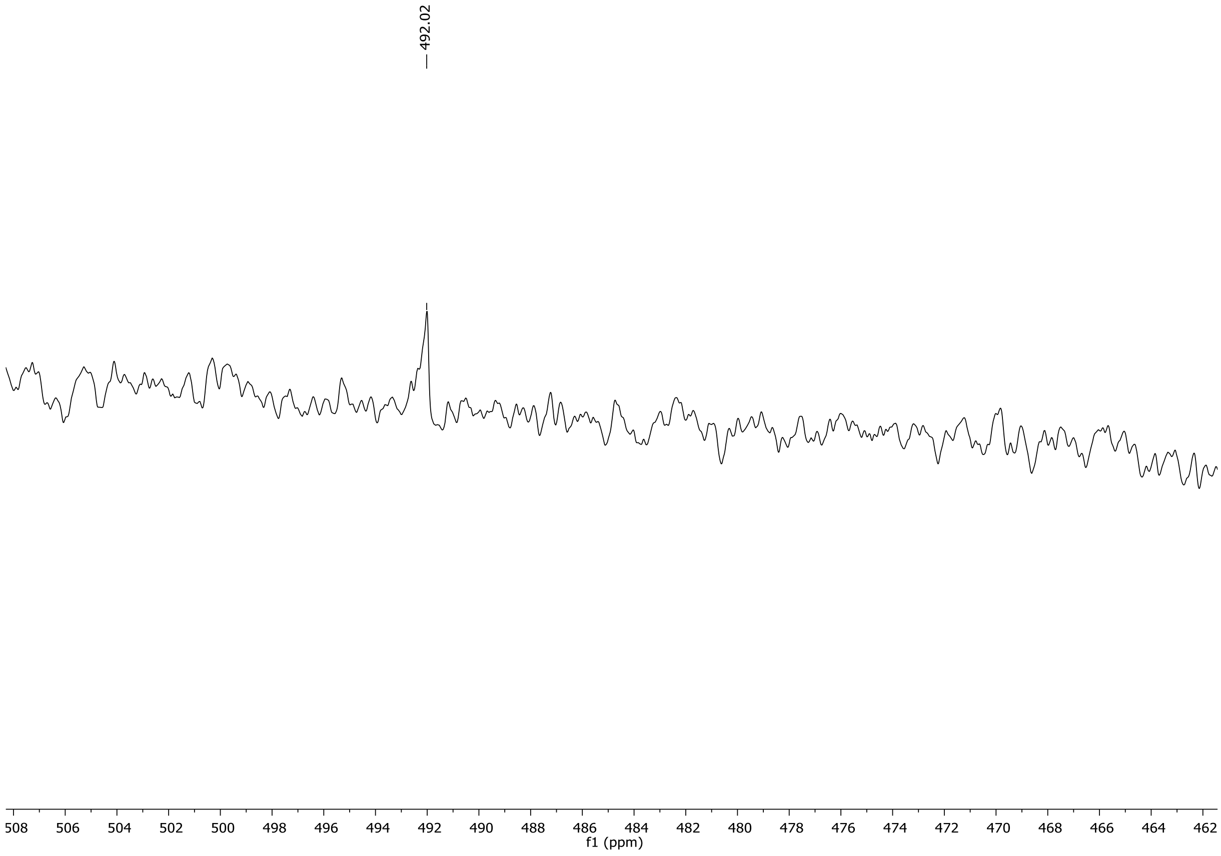

Figure S12: ^{171}Yb NMR spectrum of **1c** in THF-d_8.

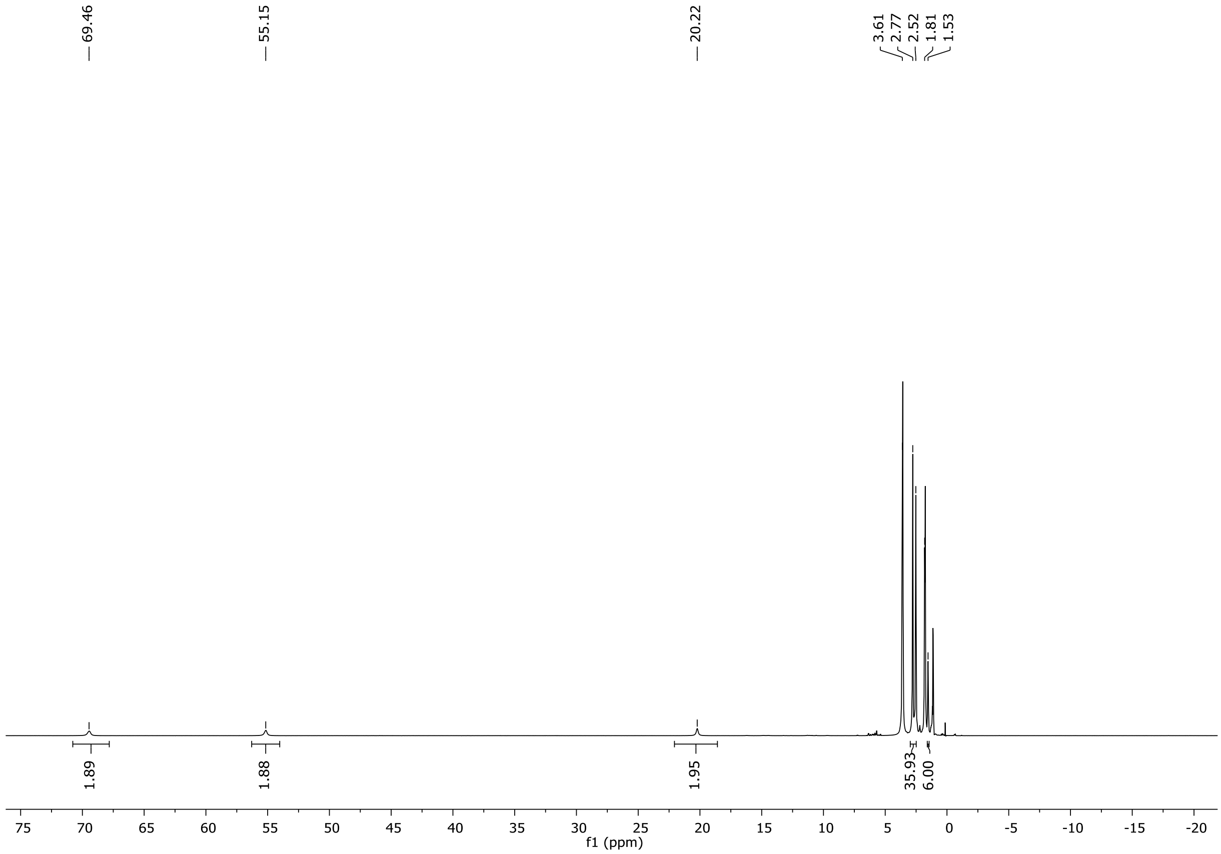

Figure S13: ^{1}H NMR spectrum of **2a** in THF-d_8. Note, compound **2a** is paramagnetic.

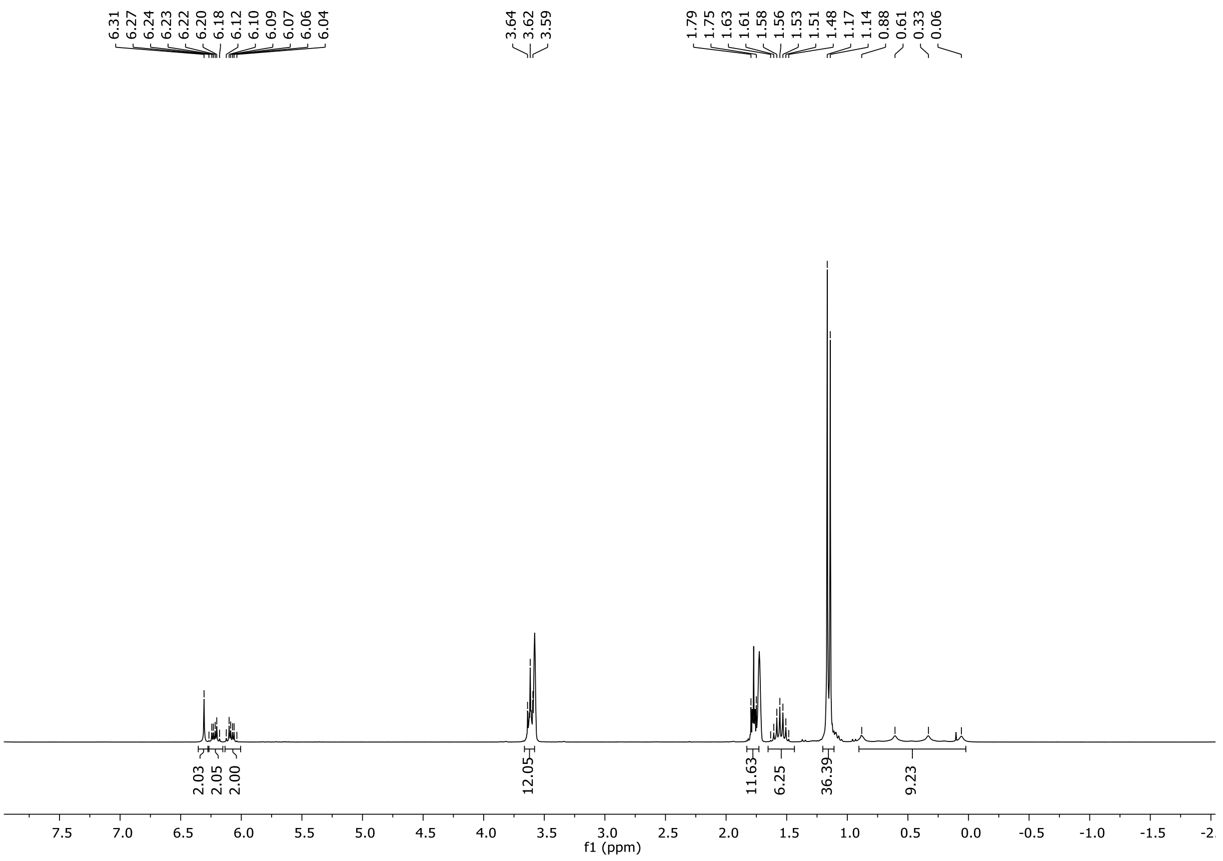

Figure S14: ^{1}H NMR spectrum of **2c** in THF-d_8.

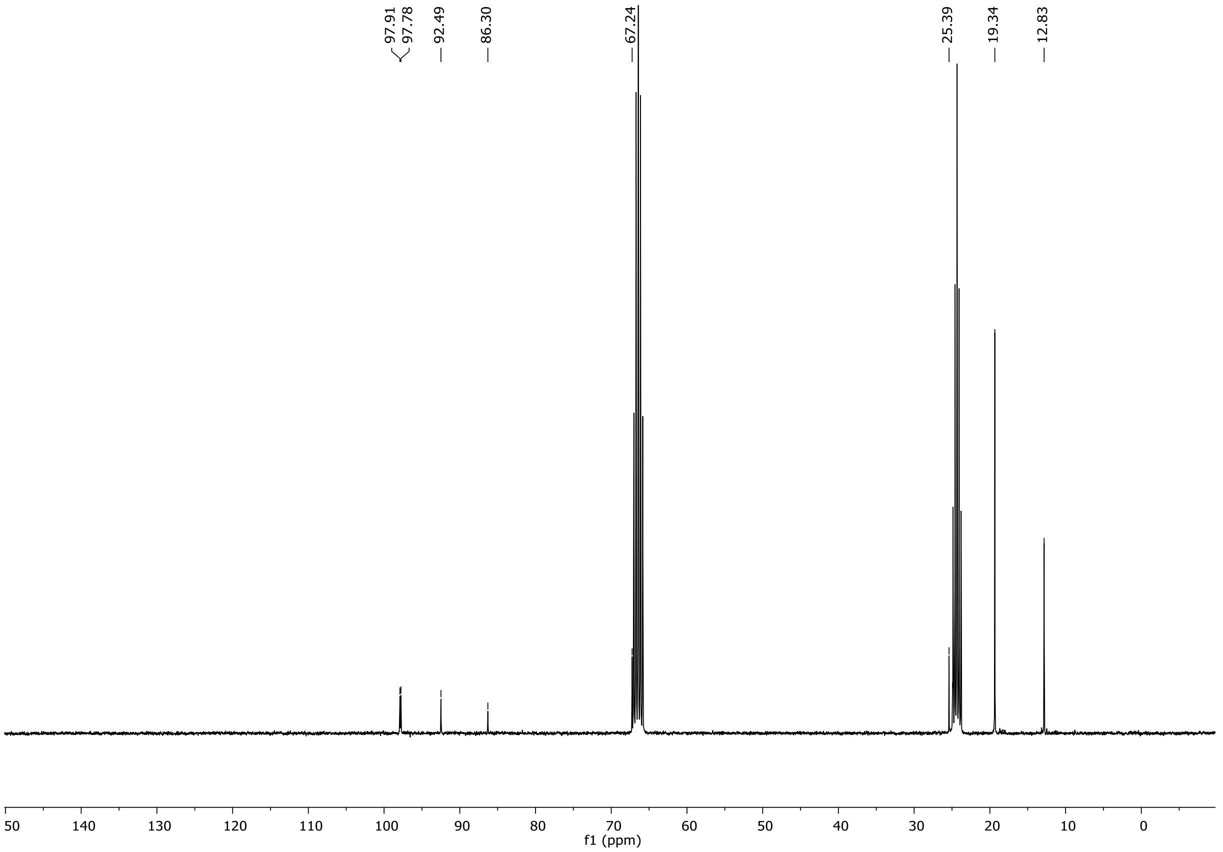

Figure S15: ^{13}C{^{1}H} NMR spectrum of **2c** in THF-d_8.

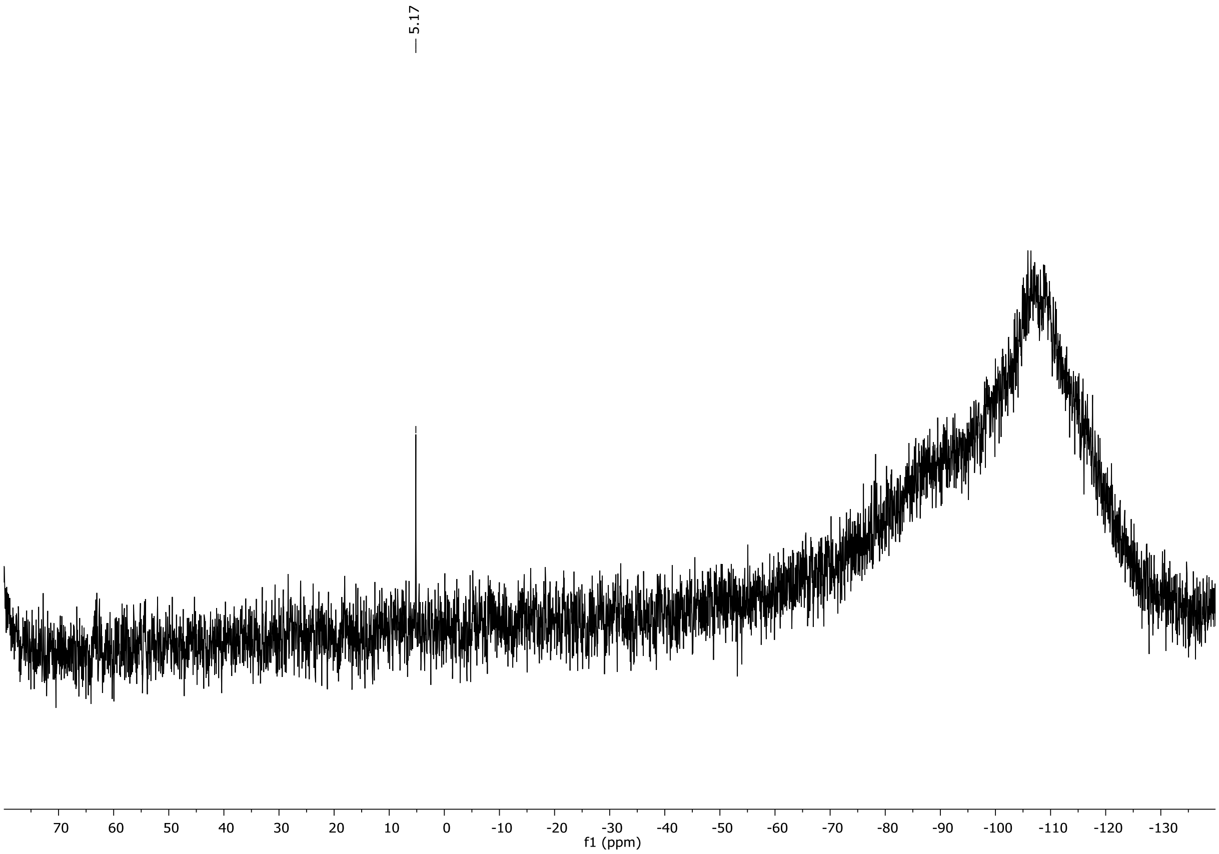

Figure S16: ^{29}Si NMR spectrum of **2c** in THF-d_8.

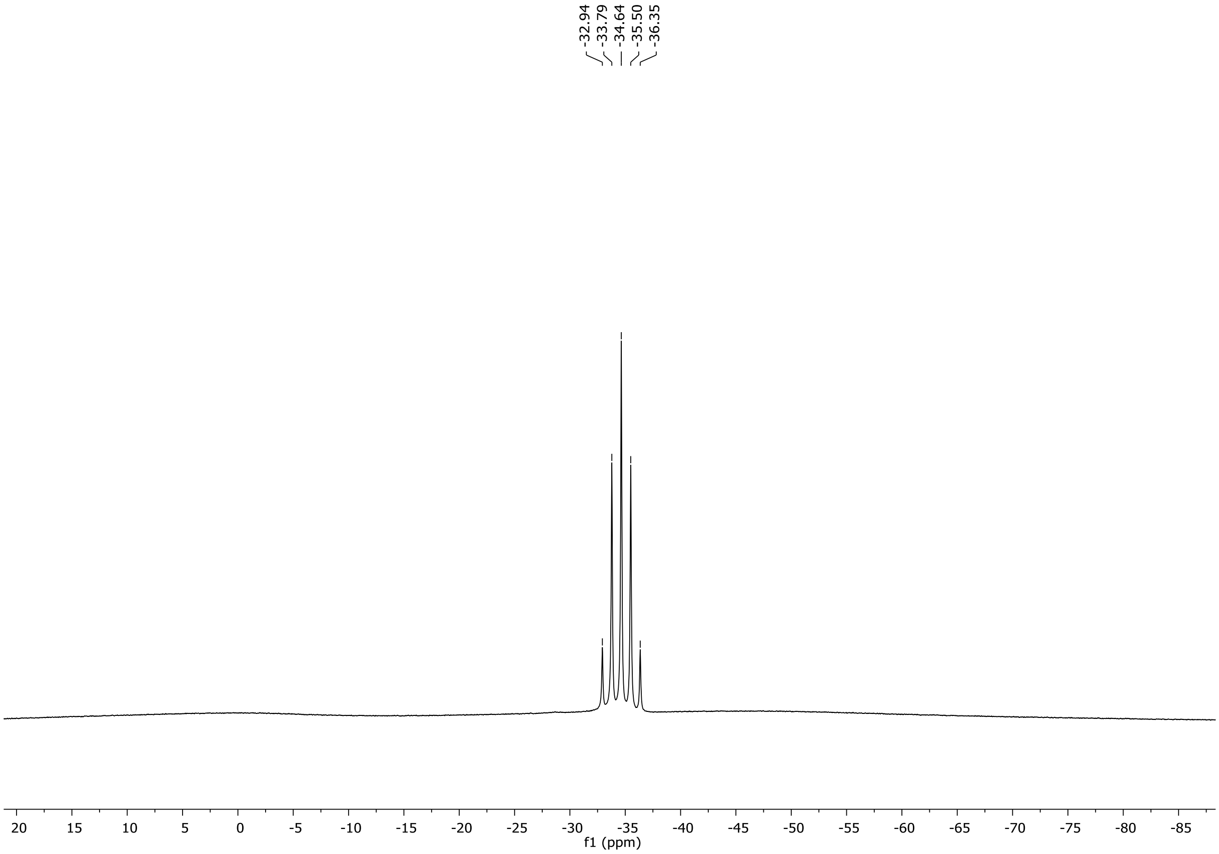

Figure S17: ^{11}B NMR spectrum of **2c** in THF-d_8.

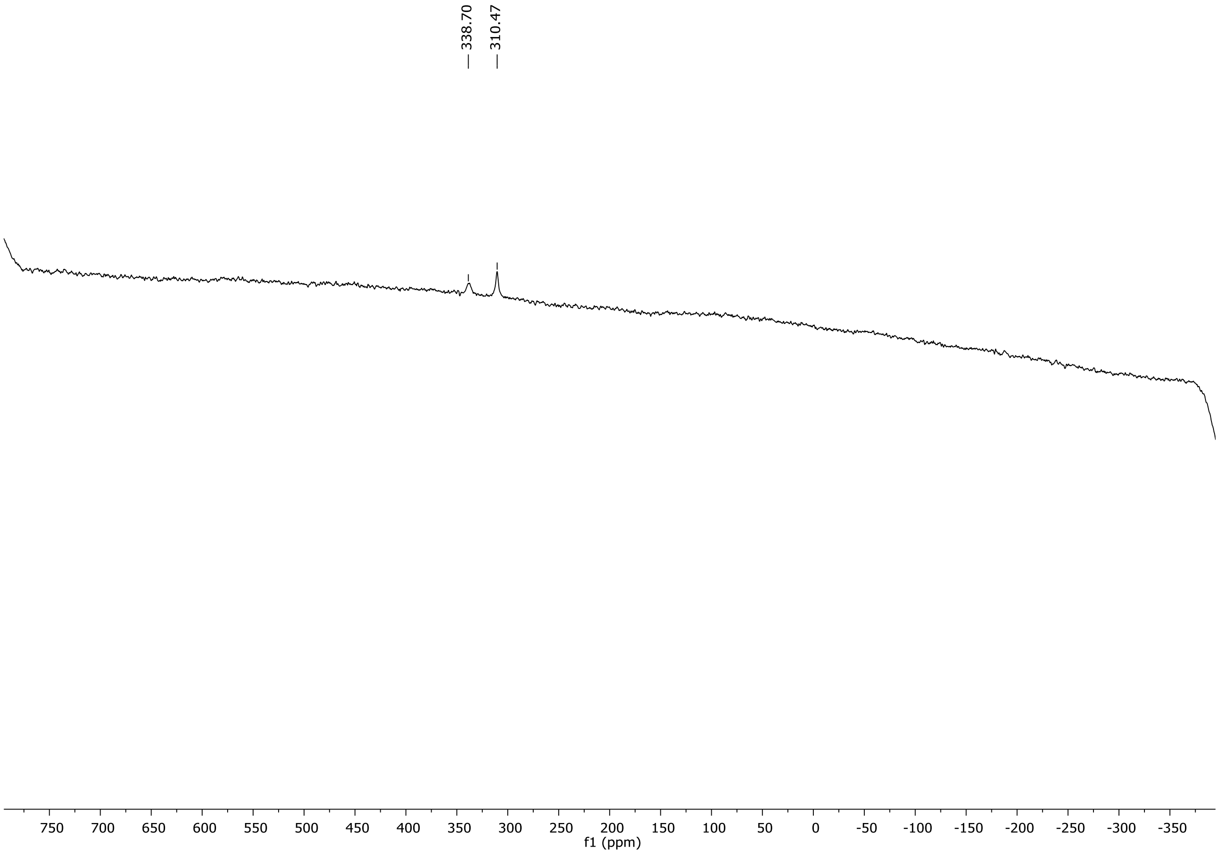

Figure S18: ^{171}Yb NMR spectrum of **2c** in THF-d_8.

6 Raman Spectra

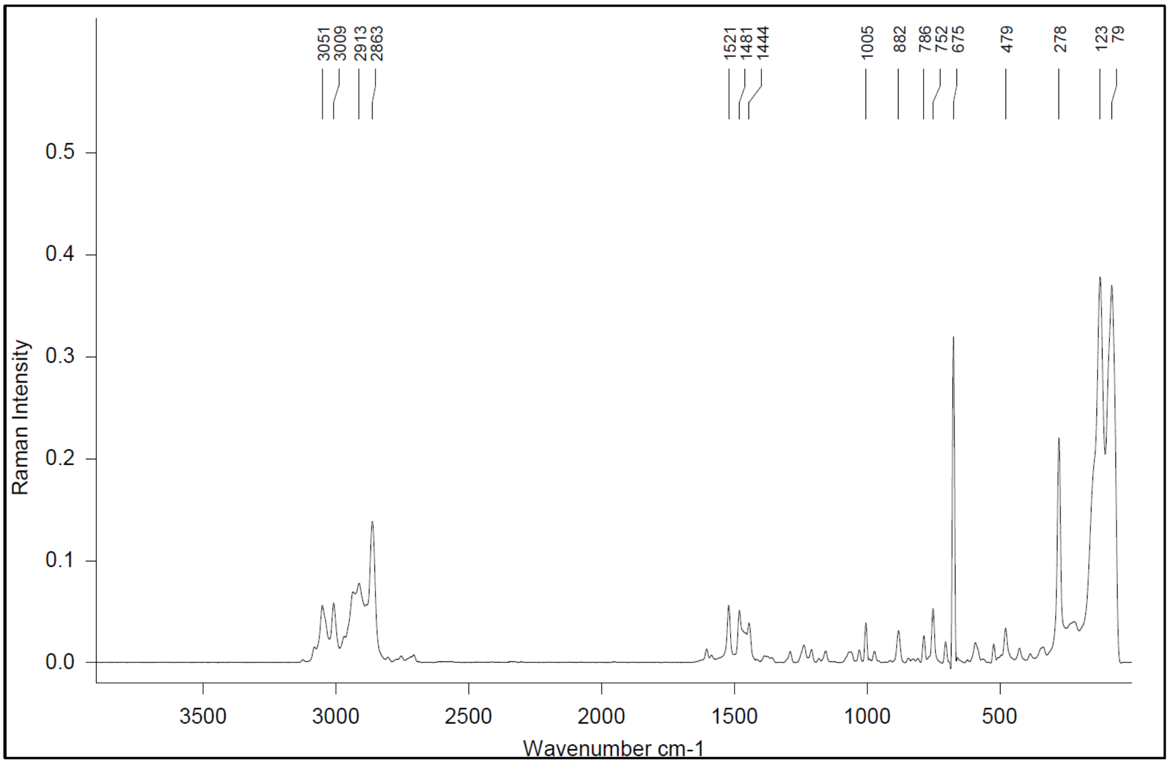

Figure S19: FT-Raman spectrum of compound **1a**.

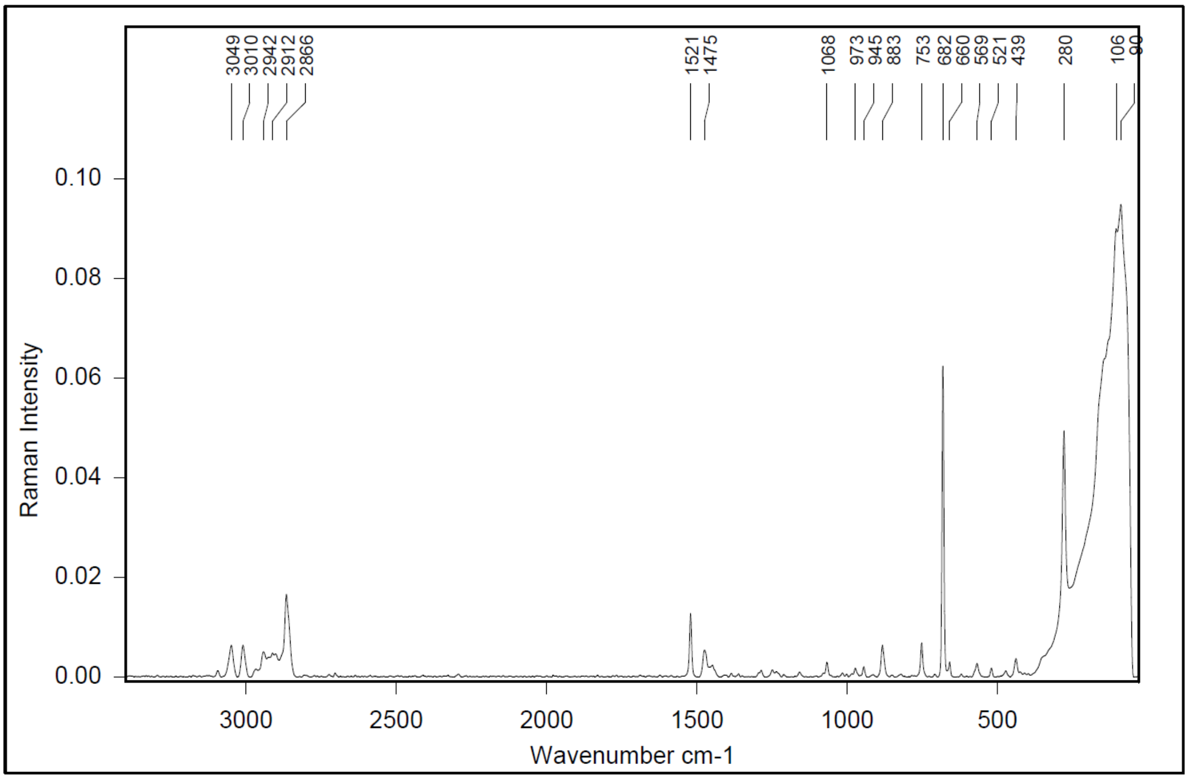

Figure S20: FT-Raman spectrum of compound **1c**.

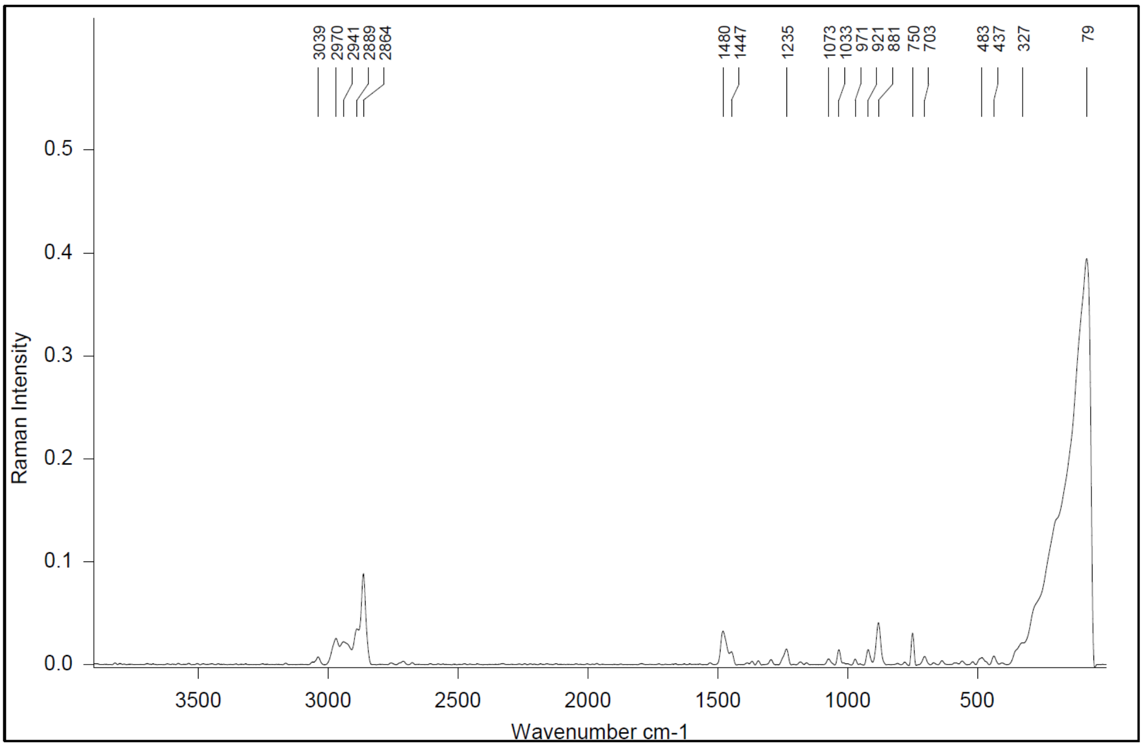

Figure S21: FT-Raman spectrum of compound **2a**.

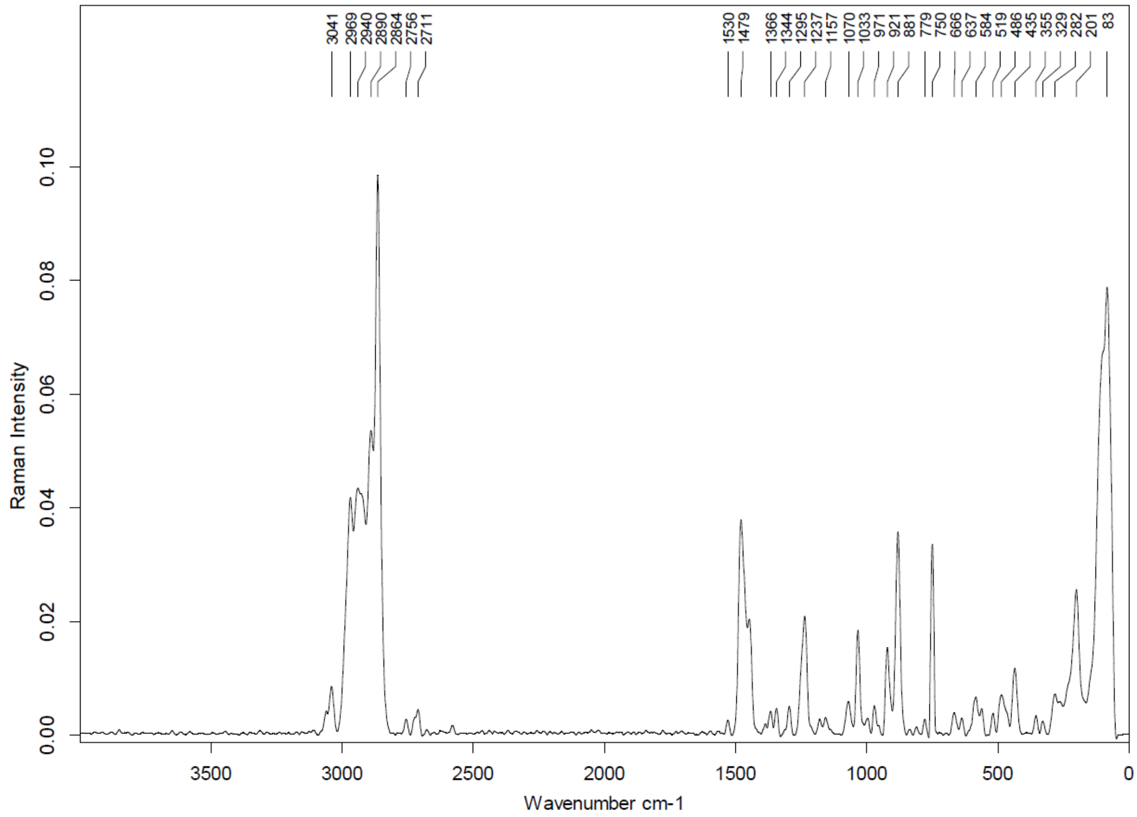

Figure S22: FT-Raman spectrum of compound **2b**.

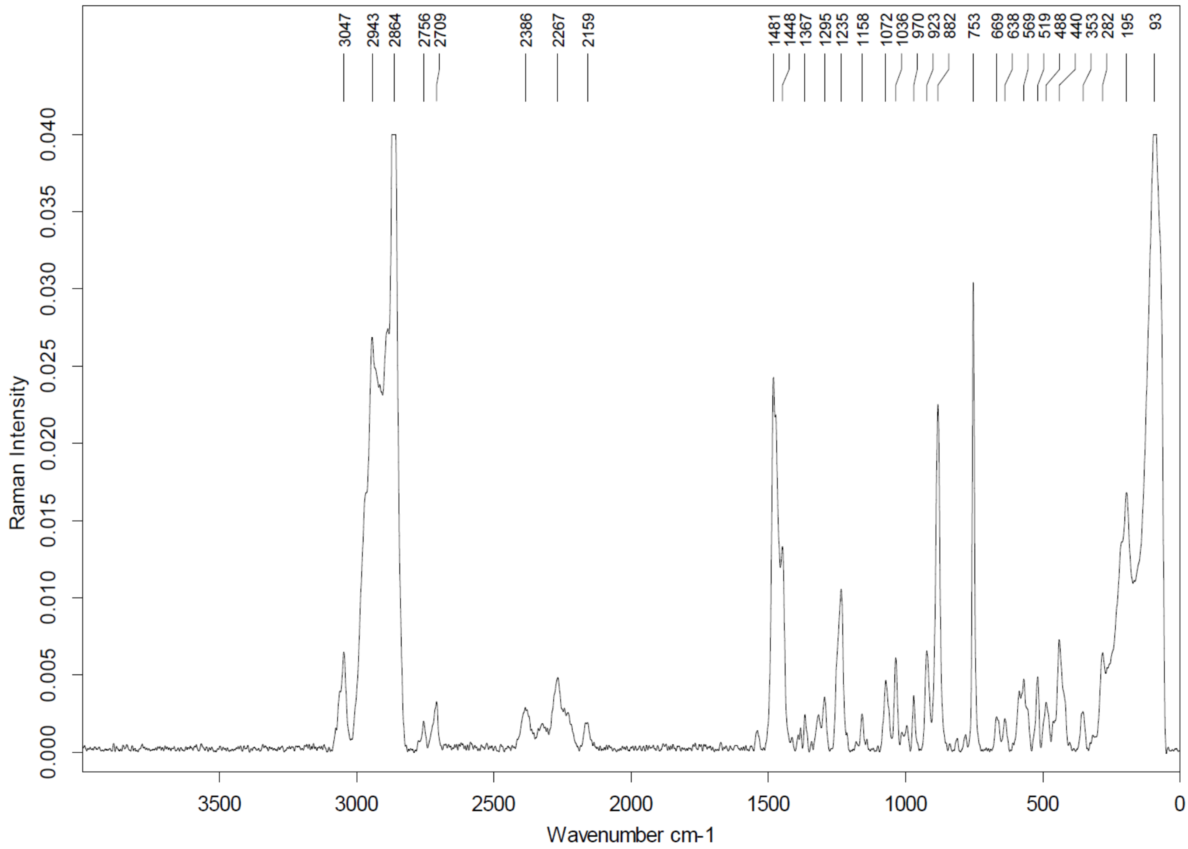

Figure S23: FT-Raman spectrum of compound **2c**.

7 IR Spectra

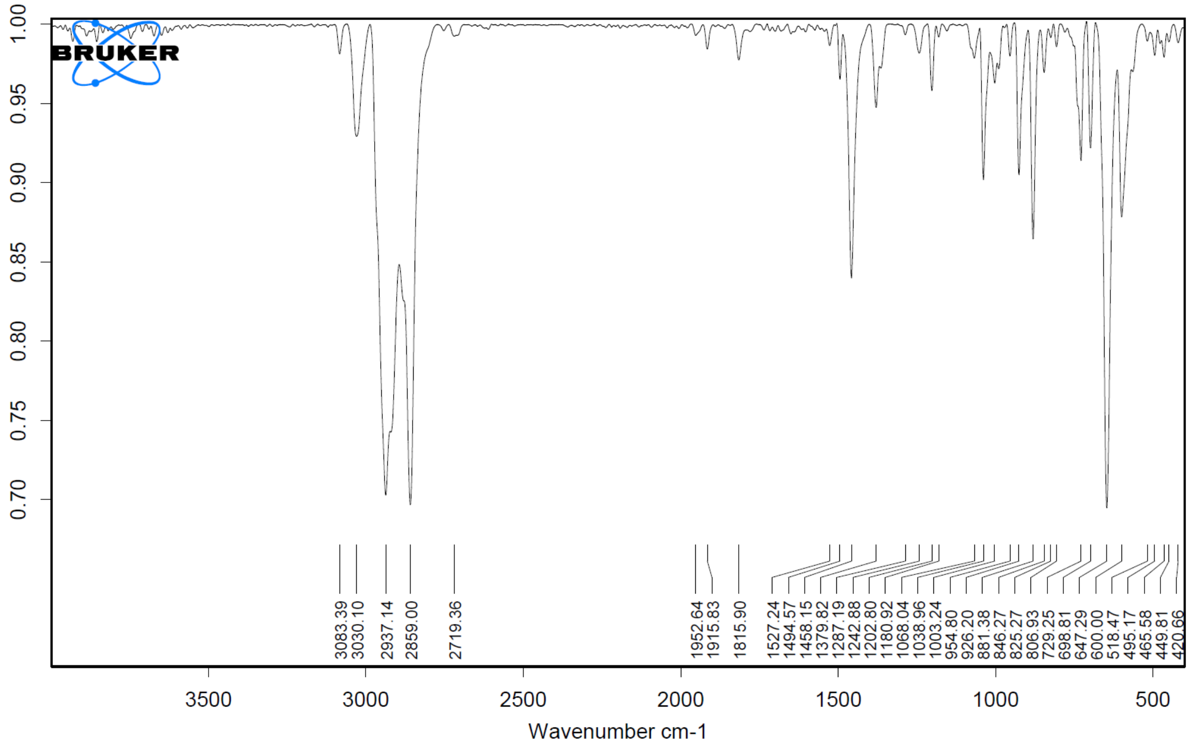

Figure S24: IR spectrum of compound **1a**.

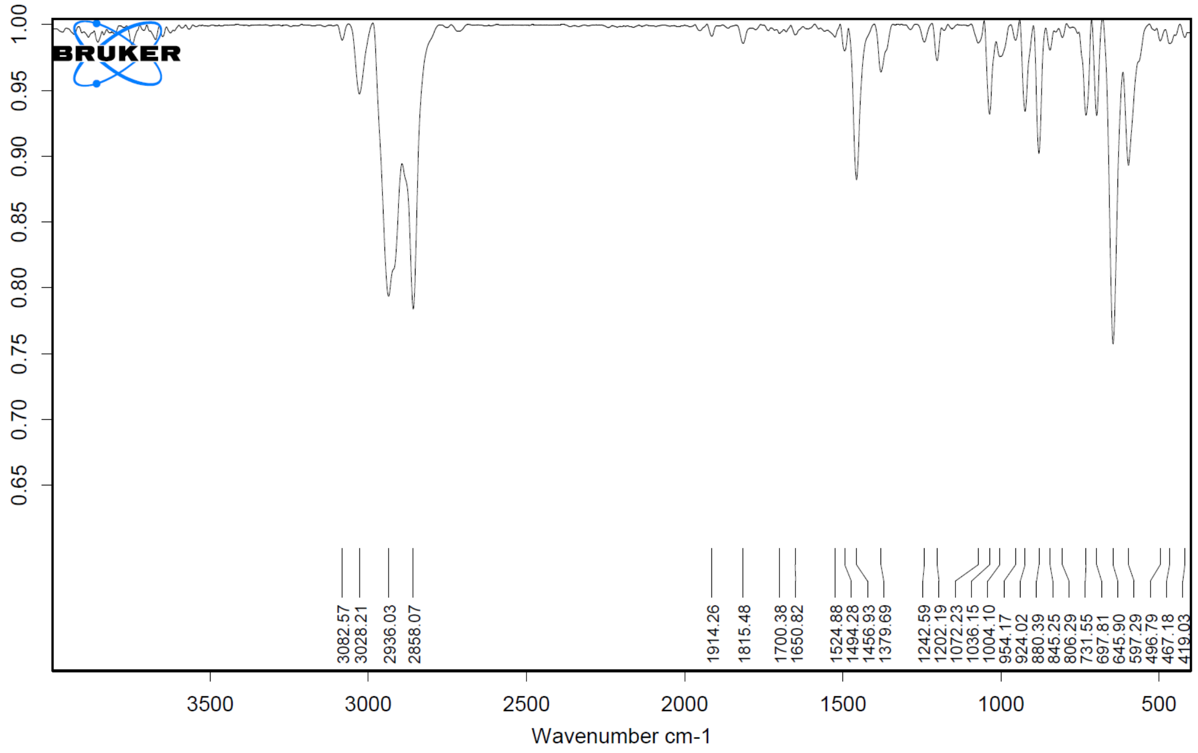

Figure S25: IR spectrum of compound **1b**.

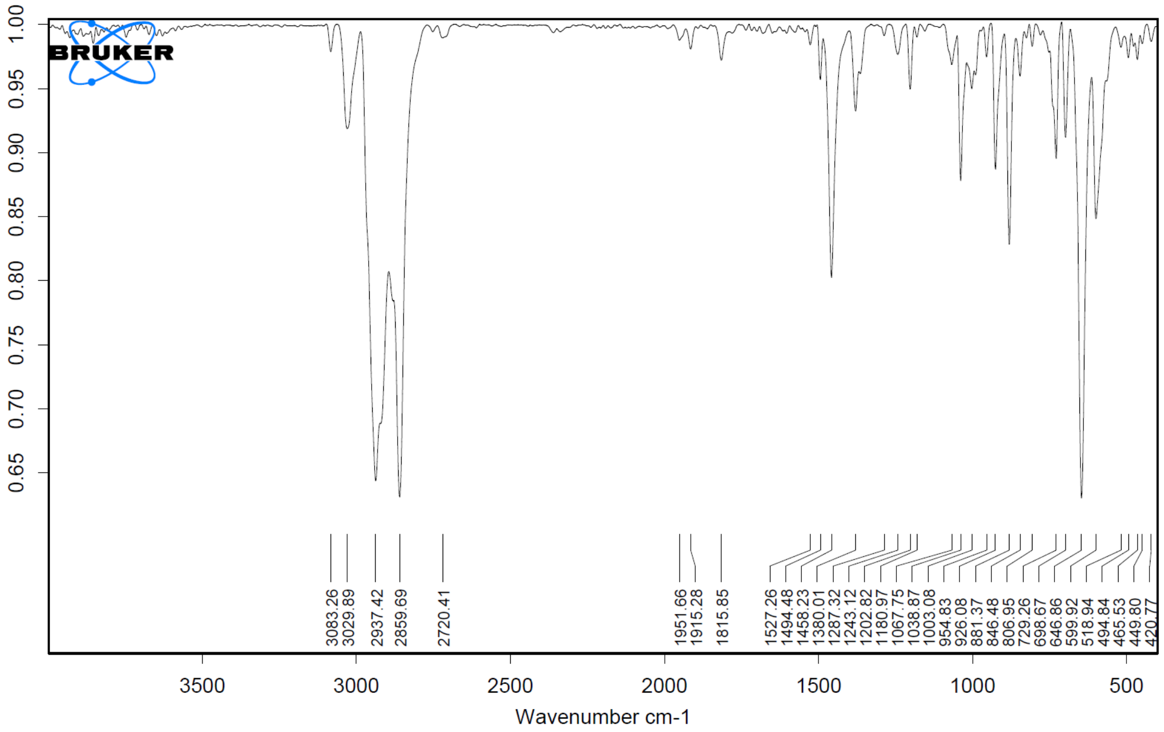

Figure S26: IR spectrum of compound **1c**.

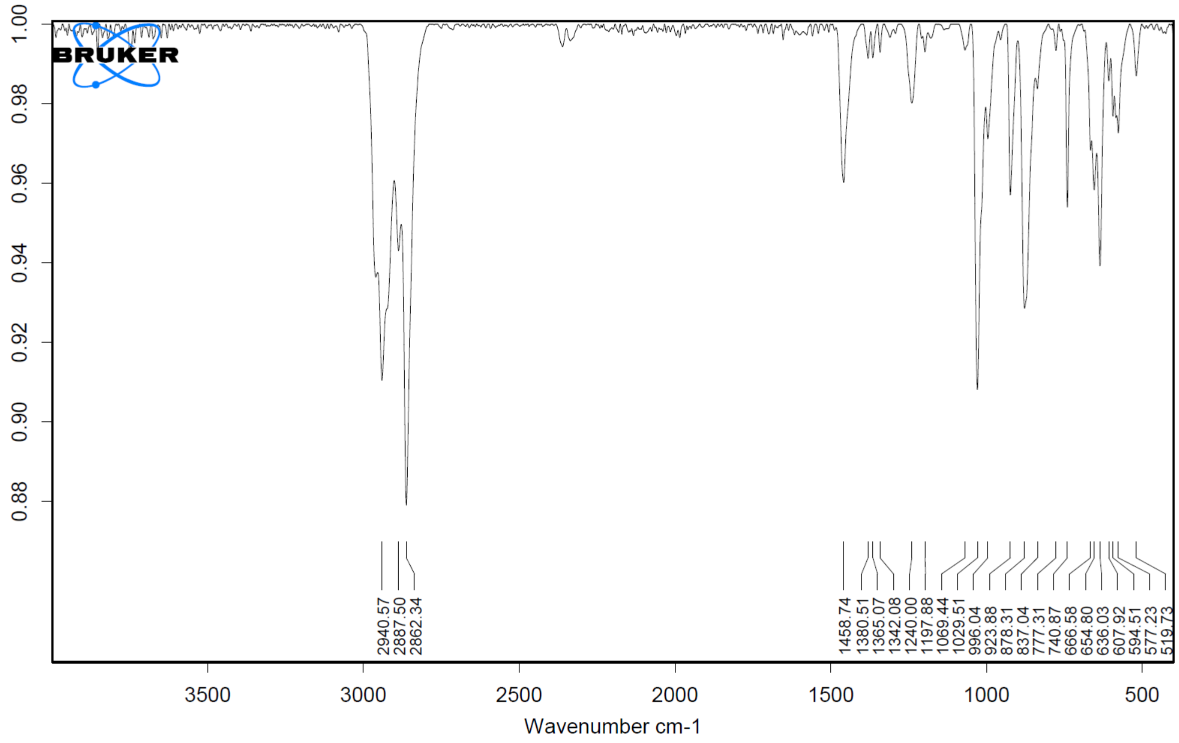

Figure S27: IR spectrum of compound **2a**.

8 Quantum Chemical Calculations

Quantum chemical calculations at the density functional theory (DFT) level were performed with TURBOMOLE.[7] Optimized structures of all compounds considered in the calculations are given in the separate ASCII file optimized-structures.txt. Unless explicitly noted otherwise, the following settings were used:

Structures were optimized with the PBE0 hybrid functional,[8] and def2-TZVP basis sets[9] together with the effective core potentials (ECP-28) for Sm, Eu and Yb were used.[10] The resolution of the identity approximation for the Coulomb part (RI-*J*)[11] in combination with the corresponding auxiliary basis sets as well as the multipole-accelerated RI-*J* approximation (MARI-*J*)[12] were employed. The D4 dispersion correction was used.[13] Self-consistent field (SCF) thresholds were set to 10^{-7} E_h, and medium sized grids (gridsize 3) were used for the numerical integration of the exchange-correlation terms.[14]

For the estimation of the dissociation energies of [$Ln^{II}(\eta^9$-Cnt$)_2$] to [$Ln^{II}(\eta^9$-Cnt)]$^+$ and Cnt^- as well as [(η^9-Cnt)$Ln^{II}(\mu$-η^9:η^9-Cnt)$Ln^{II}(\eta^8$-Cot^{TIPS})] to [$Ln^{II}(\eta^9$-Cnt)]$^+$ and [$Ln^{II}(\eta^8$-Cot^{TIPS})(η^9-Cnt)]$^-$, all relevant structures were optimized employing the conductor-like screening model (COSMO) with the dielectric constant of toluene (ε = 2.4) and otherwise default parameters.[15]

To obtain the reaction path described in the main document, the preoriented starting materials, [$Yb^{II}(\eta^8$-Cot^{TIPS})] and [$Yb^{II}(\eta^9$-Cnt$)_2$], and the final product, [(η^9-Cnt)$Yb^{II}(\mu$-η^8:η^8-Cot^{TIPS})$Yb^{II}(\eta^9$-Cnt)], were used as the input and initial guesses for intermediate structures obtained with the TURBOMOLE tool woelfling.[16] Promising candidates for minima and transitions states were subjected to further optimization, finally resulting in the identification of five intermediate local minima and five transitions states along the reaction coordinate. All calculations concerning the reaction path were performed at the PBE0/def2-SV(P) level. Tight SCF thresholds of 10^{-9} E_h and large grids (gridsize 5) were used. Again, the RI-*J* and MARI-*J* approximations as well as the D4 dispersion correction were employed. The derivatives of the quadrature weights were included in numerical integrations. Vibrational frequency calculations with the TURBOMOLE module aoforce[17] confirmed the nature of the identified intermediate structures as minima and transitions states, respectively. Optimized structures of all identified minima and transition states are given in the separate ASCII file path-structures.txt. mp4 files of the reaction path as well as the imaginary frequency vibrations of the transition states are available as additional supplementary material.

Reaction energy

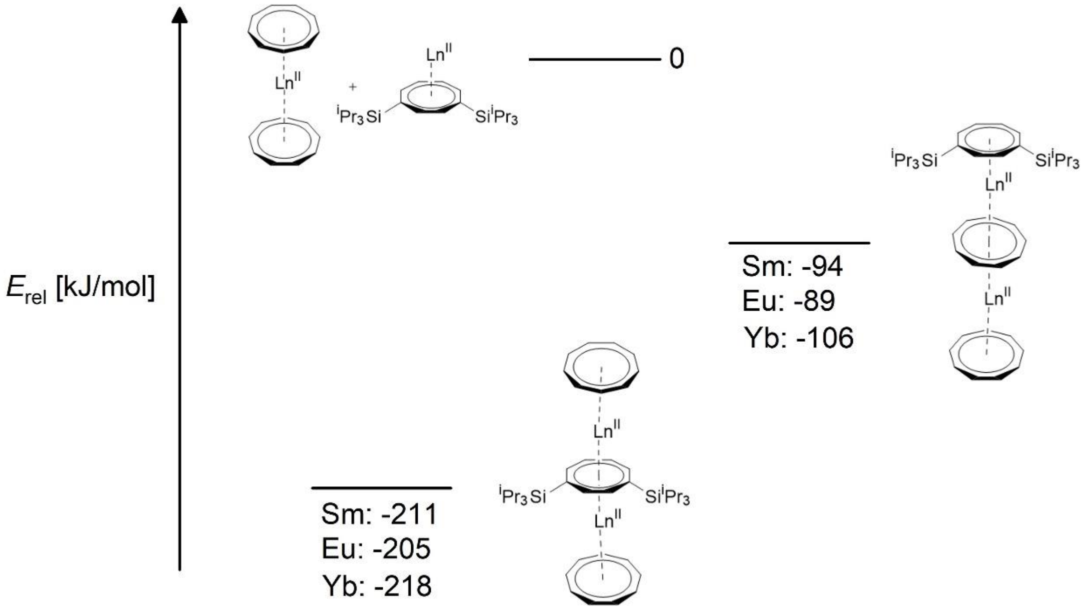

Figure S28: Energies (PBE0/def2-TZVP/D4) of the two constitutional isomers [(η^9-Cnt)Ln^{II}(μ-η^8:η^8-Cot^{TIPS})Ln^{II}(η^9-Cnt)] and [(η^9-Cnt)Ln^{II}(μ-η^9:η^9-Cnt)Ln^{II}(η^8-Cot^{TIPS})] (Ln = Sm, Eu, Yb) relative to the sum of the energies of [Ln^{II}(η^9-Cnt)$_2$] and [Ln^{II}(η^8-Cot^{TIPS})].

Estimation of Coulomb Interaction Energy

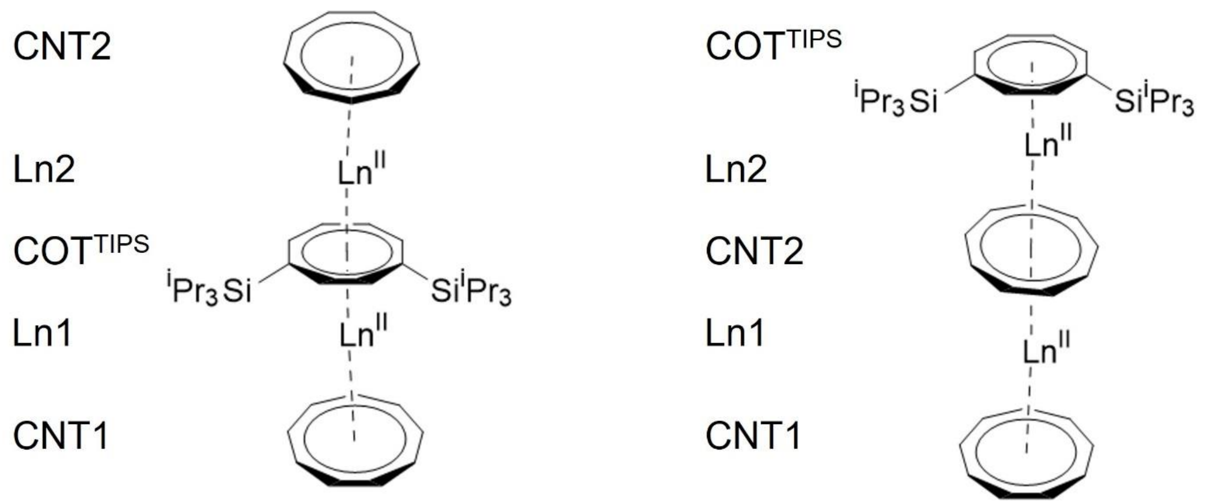

Figure S29: Labels referring to Tables S9 and S10 for [(η^9-Cnt)Ln^{II}(μ-η^8:η^8-CotTIPS)Ln^{II}(η^9-Cnt)] (left) and [(η^9-Cnt)Ln^{II}(μ-η^9:η^9-Cnt)Ln^{II}(η^8-CotTIPS)] (right).

Table S9: Natural population analysis (NPA) charges (PBE0/def2-TZVP/D4) of the Ln ions and cyclic ligands in [(η^9-Cnt)Ln^{II}(μS-η^8:η^8-Cot^{TIPS})Ln^{II}(η^9-Cnt)] and [(η^9-Cnt)Ln^{II}(μ-η^9:η^9-Cnt)Ln^{II}(η^8-Cot^{TIPS})]. Labels refer to Figure S29.

	[(η^9-Cnt)Ln^{II}(μ-η^8:η^8-Cot^{TIPS})Ln^{II}(η^9-Cnt)]			[(η^9-Cnt)Ln^{II}(μ-η^9:η^9-Cnt)Ln^{II}(η^8-Cot^{TIPS})]		
	Sm	Eu	Yb	Sm	Eu	Yb
Ln1	1.22	1.23	1.13	1.25	1.25	1.15
Ln2	1.22	1.23	1.13	1.16	1.16	1.11
Cnt1	-0.63	-0.63	-0.57	-0.56	-0.56	-0.50
Cnt2	-0.63	-0.63	-0.57	-0.64	-0.63	-0.57
CotTIPS	-1.19	-1.19	-1.11	-1.21	-1.22	-1.19

Table S10: Selected distances (in a.u.) within [(η^9-Cnt)Ln^{II}(μ-η^8:η^8-Cot^{TIPS})Ln^{II}(η^9-Cnt)] and [(η^9-Cnt)Ln^{II}(μ-η^9:η^9-Cnt)Ln^{II}(η^8-Cot^{TIPS})] (PBE0/def2-TZVP/D4). For the cyclic ligands, the distances refer to the ring centroid. Labels refer to Figure S29.

	[(η^9-Cnt)Ln^{II}(μ-η^8:η^8-Cot^{TIPS})Ln^{II}(η^9-Cnt)]			[(η^9-Cnt)Ln^{II}(μ-η^9:η^9-Cnt)Ln^{II}(η^8-Cot^{TIPS})]		
	Sm	Eu	Yb	Sm	Eu	Yb
Cnt1-Ln1	4.00	4.02	3.74	3.81	3.83	3.50
Cnt1-CotTIPS	8.01	8.02	7.52	16.45	16.52	15.49
Cnt1-Ln2	12.02	12.03	11.30	13.05	13.12	12.28
Cnt1-Cnt2	15.99	16.01	14.99	7.88	7.92	7.39
Ln1-CotTIPS	4.018	4.02	3.79	12.75	12.80	12.14
Ln1-Ln2	8.03	8.03	7.59	9.31	9.37	8.91
Ln1-Cnt2	12.02	12.03	11.30	4.07	4.08	3.89
CotTIPS-Ln2	4.016	4.01	3.79	3.62	3.61	3.33
CotTIPS-Cnt2	8.01	8.02	7.52	8.87	8.92	8.55
Ln2-Cnt2	4.01	4.02	3.74	5.40	5.46	5.34

Based on the data given in Tables S9 and S10, the electrostatic contribution to the total energy within the complexes [(η^9-Cnt)Ln^{II}(μ-η^8:η^8-Cot^{TIPS})Ln^{II}(η^9-CSNT)] and [(η^9-Cnt)Ln^{II}(μ-η^9:η^9-Cnt)Ln^{II}(η^8-Cot^{TIPS})] can be estimated in a simplified manner based on Coulomb´s law:

$$E_{\mathrm{C}}= \frac{Q_{Ln1}*Q_{Ln2}}{r_{Ln1-Ln2}} + \frac{Q_{CNT1}*Q_{CNT2}}{r_{CNT1-CNT2}} + \frac{Q_{CNT1}*Q_{COTTIPS}}{r_{CNT1-COTTIPS}} + \frac{Q_{CNT2}*Q_{COTTIPS}}{r_{CNT2-COTTIPS}} - \frac{Q_{CNT1}*Q_{Ln1}}{r_{CNT1-Ln1}} - \frac{Q_{CNT1}*Q_{Ln2}}{r_{CNT1-Ln2}} - \frac{Q_{CNT2}*Q_{Ln1}}{r_{CNT2-Ln1}} - \frac{Q_{CNT2}*Q_{Ln2}}{r_{CNT2-Ln2}} - \frac{Q_{COTTIPS}*Q_{Ln1}}{r_{COTTIPS-Ln1}} - \frac{Q_{COTTIPS}*Q_{Ln2}}{r_{COTTIPS-Ln2}}$$

Differences in electrostatic interaction energy between the two constitutional isomers [(η^9-Cnt)Ln^{II}(μ-η^8:η^8-Cot^{TIPS})Ln^{II}(η^9-Cnt)] and [(η^9-Cnt)Ln^{II}(μ-η^9:η^9-Cnt)Ln^{II}(η^8-Cot^{TIPS})] derived this way are given within the main text.

Reaction path

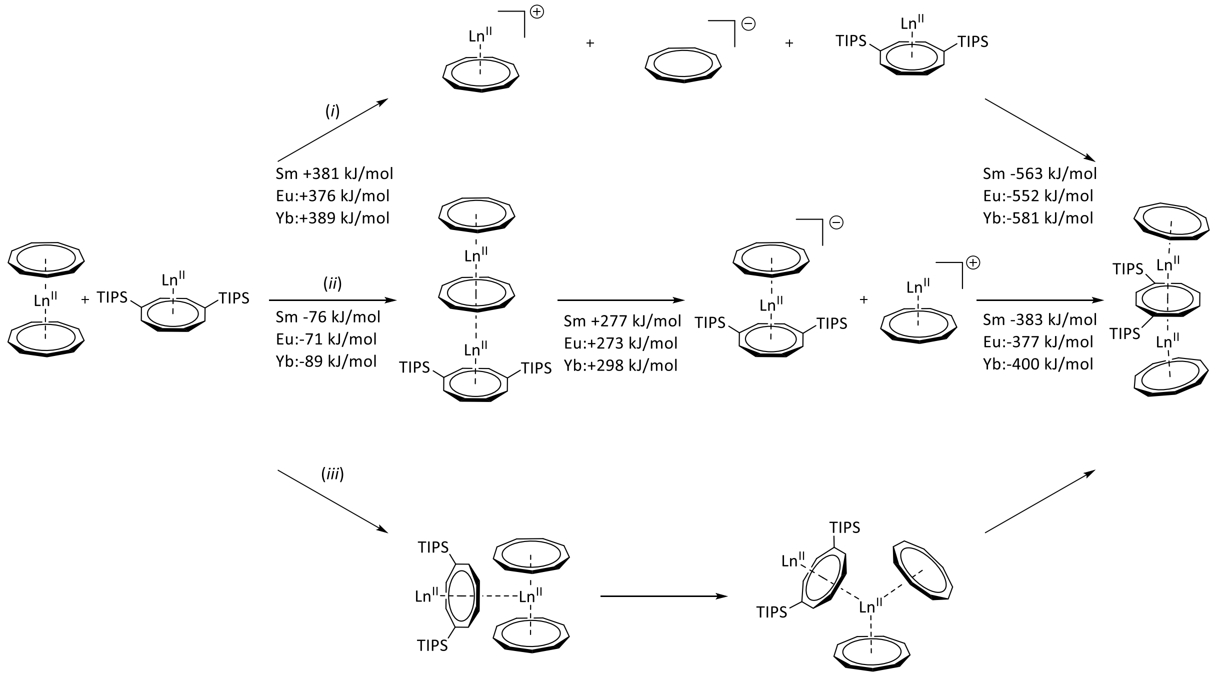

Figure S30: Schematic illustration of plausible formation mechanisms of [(η^9-Cnt)Ln^{II}(μ-η^8:η^8-Cot^{TIPS})Ln^{II}(η^9-Cnt)] from [Ln^{II}(η^9-Cnt)$_2$] and [Ln^{II}(η^8-Cot^{TIPS})] discussed in detail in the main document. For pathways (*i*) and (*ii*), given energy differences refer to calculations at the PBE0/def2-TZVP/D4/COSMO(ε=2.4) level of theory. For pathway (*iii*), a reaction path optimization was performed for Ln = Yb at the PBE0/def2-SV(P)/D4 level (refer to Figure 5 in the main document and computational details above).

9 References

[1] a) T. J. Katz, P. J. Garratt, *J. Am. Chem. Soc.* **1964**, *86*, 5194; b) L. Münzfeld, C. Schoo, S. Bestgen, E. Moreno-Pineda, R. Köppe, M. Ruben, P. W. Roesky, *Nat. Comm.* **2019**, *10*, 3135.

[2] O. T. Summerscales, F. G. N. Cloke, P. B. Hitchcock, J. C. Green, N. Hazari, *Science* **2006**, *311*, 829.

[3] a) K. Kawasaki, R. Sugiyama, T. Tsuji, T. Iwasa, H. Tsunoyama, Y. Mizuhata, N. Tokitoh, A. Nakajima, *ChemComm* **2017**, *53*, 6557; b) M. Xémard, S. Zimmer, M. Cordier, V. Goudy, L. Ricard, C. Clavaguéra, G. Nocton, *J. Am. Chem. Soc.* **2018**, *140*, 14433.

[4] G. Sheldrick, *Acta Crystallogr. Sect. A* **2015**, *71*, 3.

[5] G. Sheldrick, *Acta Crystallogr. Sect. C* **2015**, *71*, 3.

[6] O. V. Dolomanov, L. J. Bourhis, R. J. Gildea, J. A. K. Howard, H. Puschmann, *J. Appl. Crystallogr.* **2009**, *42*, 339.

[7] a) *TURBOMOLE V7.8 2023 , a development of University of Karlsruhe and Forschungszentrum Karlsruhe GmbH, 1989−2007, TURBOMOLE GmbH, since 2007; available from* *https://www.turbomole.org*; b) Y. J. Franzke, C. Holzer, J. H. Andersen, T. Begušić, F. Bruder, S. Coriani, F. Della Sala, E. Fabiano, D. A. Fedotov, S. Fürst, S. Gillhuber, R. Grotjahn, M. Kaupp, M. Kehry, M. Krstić, F. Mack, S. Majumdar, B. D. Nguyen, S. M. Parker, F. Pauly, A. Pausch, E. Perlt, G. S. Phun, A. Rajabi, D. Rappoport, B. Samal, T. Schrader, M. Sharma, E. Tapavicza, R. S. Treß, V. Voora, A. Wodyński, J. M. Yu, B. Zerulla, F. Furche, C. Hättig, M. Sierka, D. P. Tew, F. Weigend, *J. Chem. Theory Comput.* **2023**, *19*, 6859.

[8] a) J. P. Perdew, M. Ernzerhof, K. Burke, *J. Chem. Phys.* **1996**, *105*, 9982; b) C. Adamo, V. Barone, *J. Chem. Phys.* **1999**, *110*, 6158.

[9] F. Weigend, R. Ahlrichs, *PCCP* **2005**, *7*, 3297.

[10] M. Dolg, H. Stoll, H. Preuss, *J. Chem. Phys.* **1989**, *90*, 1730.

[11] F. Weigend, *PCCP* **2006**, *8*, 1057.

[12] M. Sierka, A. Hogekamp, R. Ahlrichs, *J. Chem. Phys.* **2003**, *118*, 9136.

[13] E. Caldeweyher, C. Bannwarth, S. Grimme, *J. Chem. Phys.* **2017**, *147*, 034112.

[14] O. Treutler, R. Ahlrichs, *J. Chem. Phys.* **1995**, *102*, 346.

[15] A. Schäfer, A. Klamt, D. Sattel, J. C. W. Lohrenz, F. Eckert, *PCCP* **2000**, *2*, 2187.

[16] P. Plessow, *J. Chem. Theory Comput.* **2013**, *9*, 1305.

[17] a) P. Deglmann, F. Furche, R. Ahlrichs, *Chem. Phys. Lett.* **2002**, *362*, 511; b) P. Deglmann, K. May, F. Furche, R. Ahlrichs, *Chem. Phys. Lett.* **2004**, *384*, 103; c) P. Deglmann, F. Furche, *J. Chem. Phys.* **2002**, *117*, 9535.
